# Improving the Calibration of Surface Time Domain Reflectometry Sensors for Assessing the Moisture of Building Materials Taking into Account Signal Attenuation

**DOI:** 10.3390/ma19183891

**Published:** 2026-09-12

**Authors:** Zbigniew Suchorab, Magdalena Jastrzębska, Anna Futa, Magdalena Paśnikowska-Łukaszuk, Magda Wlazło, Krzysztof Tabiś

**Affiliations:** 1 Department of Water Supply and Wastewater Disposal, Faculty of Environmental Engineering, Lublin University of Technology, Nadbystrzycka 40B, 20-618 Lublin, Poland; 2 Department of Applied Mathematics, Faculty of Mathematics and Information Technology, Lublin University of Technology, Nadbystrzycka 38, 20-618 Lublin, Poland; m.jastrzebska@pollub.pl (M.J.); a.futa@pollub.pl (A.F.); 3 Department of Technical Computer Science, Faculty of Mathematics and Information Technology, Lublin University of Technology, Nadbystrzycka 38, 20-618 Lublin, Poland; m.pasnikowska-lukaszuk@pollub.pl (M.P.-Ł.); m.wlazlo@pollub.pl (M.W.); 4 Aquapol Polska CPV Research Laboratory Krzysztof Tabiś, Stefana Żeromskiego 12, 58-160 Świebodzice, Poland; ktabis@aquapol.pl

**Keywords:** Time Domain Reflectometry, moisture, salinity, apparent permittivity, signal attenuation, multiple regression

## Abstract

**Highlights:**

• Improving the calibration of a noninvasive TDR sensor for moisture detection.
• Taking into account signal attenuation for the calibration of reflectometric sensors to determine the moisture of porous materials.
• Taking into account the influence of salinity on material moisture readings without the need to mark its level.
• Application of multiple regression to determine the moisture content of saline porous materials using the TDR method.

**Abstract:**

The article describes the problem of moisture in buildings and building materials as well as the Time Domain Reflectometry (TDR) measurement technique that can be applied for invasive and also noninvasive measurements of building materials. Moisture readings in such media rely on signal propagation time shift analysis and may be subject to errors, while the vast majority of models derived for TDR devices are based on this type of measurement. Multiple linear regression (MLR) is proposed as a method to improve the quality of the readouts achieved using TDR noninvasive sensors. The aim of this work was to improve the calibration methods of TDR surface sensors by taking into account the attenuation of the electromagnetic pulse caused by the presence of water and salt ions and to develop calibration formulas that are multiple linear regression equations considering both the time shift and signal attenuation. Within the research, two types of noninvasive TDR sensors were applied to measure the moisture of red ceramic, clinker and silicate bricks. Average RMSE values for all sensors and materials were 0.009 cm^3^/cm^3^ lower in the case of the proposed multiple regression calibration models and 0.005 cm^3^/cm^3^ in terms of RSE compared to the traditional calibration methods used in the TDR technique.

## 1. Introduction

Moisture is one of the most common causes of problems in building envelopes [1,2,3].

Recent studies show that moisture damage affects a share of buildings; about one-third of buildings in Sweden and one-quarter in Finland have been reported to suffer moisture damage [4,5]. In Norway, an analysis of 175 cases from 2017 to 2020 found that almost three-quarters of the defects were caused by moisture and more than seventy per cent of all defects involved the building envelope [6]. The cost side is huge. These findings prove that checking and monitoring moisture in building materials is essential not only for keeping buildings strong and well-balanced in temperature but also for slashing the money spent on early breakdown and repairs.

The basic factor influencing the formation of moisture in a wall is water, which may have several sources [7]. Water may be present within walls in three distinct forms: chemically bound water, free water vapor or free water [8]. It can also take the form of sorption moisture [9]. Capillary water reduces the thermal parameters of the building, while condensation water promotes the formation of moisture [10,11]. Moisture may also significantly affect the performance of building materials. In concrete, increasing water saturation has been shown to cause a linear decrease in compressive strength, splitting tensile strength and fracture toughness. Depending on the water-to-cement ratio, the respective reductions in saturated specimens compared with unsaturated ones reached approximately 34–40%, 35–45% and 47–57% [12]. A moisture-dependent deterioration of properties has also been observed in lignocellulosic materials, where increasing relative humidity resulted in a decrease in the Young’s modulus of lignocellulosic polymers [13]. These findings show how crucial it is to assess moisture content when evaluating the condition and performance of building materials.

The way moisture behaves in building materials hinges largely on their internal structure and physical properties. Porous materials, such as red ceramic bricks, silicate bricks and autoclaved aerated concrete, vary in pore structure, density and capillary transport [14]. These differences influence how moisture accumulates and how it distributes inside the material and matter especially when indirect moisture measurements are used. The electrical signal observed depends not only on the moisture content but also on the substrate properties, so assessment methods should consider material-specific characteristics and require calibration for various building materials [15,16].

To assess environmental parameters in buildings, various measurement methods are used, i.e., as described in ref. [17], indirect and direct. Indirect methods allow for determining the physical properties of building materials that may also be exposed to moisture [18,19]. These methods include electric and microwave methods. One such method is reflectometry, e.g., TDR (Time Domain Reflectometry) [20,21]. Application of the Time Domain Reflectometry technique enables assessment of the moisture level from 35 up to 60% mass, and the measurement uncertainty ranges from 1% mass to 10% mass depending on the degree of material moisture [22]. The propagation time measured by the TDR meter depends on the pulse propagation velocity, which in turn depends on the electrical properties of the environment—the electrical permittivity of the medium [23,24,25].

This method has been widely employed for many years to measure soil moisture [26,27]. For many years, it has been successfully applied in agriculture to monitor moisture changes in soils, which is significant in the preparatory process, e.g., [28,29,30]. Many successful initiatives have been undertaken over the last decade to use its measurement potential to determine the moisture content of materials and building partitions [31]. The TDR method is widely used to evaluate the moisture of porous materials [32,33]. The dielectric permittivity of a porous material is determined using the following formula [34,35]:

(1) ε = [(c⋅t_p_)/2L]^2^,

where: ε is the apparent permittivity of the measured material [-], c is the speed of light in vacuum [3 · 10^8^ m · s^−1^], t_p_ is the propagation time of the electromagnetic wave along the measured element and L is the length of the measured element.

The TDR technique employs two types of pulses generated by pulse generators: step pulses [36] and needle pulses [37]. Measurements obtained by a TDR multimeter from the detection sensor are represented as a series of voltage values recorded over time, referred to as reflectograms or waveforms [38]. Knowing the geometric structure of the measuring element, it is possible to determine the reflection points and thus the time of pulse propagation along the sensor [39]. This is the basis for estimating the real part of the total complex electrical permittivity, which indicates the moisture of the tested medium [40].

Very often, the tested media, both soil and building materials, are loaded with salt ions, which suppress the electromagnetic pulse that is related to ionic conductivity and affects the imaginary part of the apparent permittivity of the examined materials [25]. The permittivity ε_ω_ of the environment with non-zero electrical conductivity can be expressed using the Debye [41] or Cole–Cole [42] models. The Debye model describes apparent permittivity as a frequency function, with the real part (ε′) and the imaginary part (ε″) described with the following formulas [43]:

(2) ε′ = ε_∞_ + [(ε_s_ − ε_∞_)/(1 + ω^2^τ^2^)],

(3) ε″ = [(ε_s_ − ε_∞_)ωτ/(1 + ω^2^τ^2^)]

where: ε_∞_—permittivity at the high-frequency limit; ε_s_—static, low-frequency permittivity; τ—characteristic relaxation time of the medium; and ω—frequency of the externally applied field [s^−1^].

The Cole–Cole model represents a generalized form of the Debye dielectric relaxation model and provides a good fit to experimental measurements in numerous applications [41]. The Cole–Cole model is often applied to describe dielectric relaxation in polymers [41,43,44]. The most commonly used representation is formulated in the frequency domain, where the impedance varies as a function of frequency [41]. The Cole–Cole model is usually utilized as a useful formula to combine the observed frequency-dependent signal attenuation with velocity dispersion spectra. Formula (4) presents the equation of the Cole–Cole model:

(4) ε*(ω) = ε_∞_ + [(ε_s_ − ε_∞_)/(1 + (i ωτ)^1−α^)]

where: ε*—complex dielectric constant, ε_s_—static dielectric constant, ε_∞_—infinite frequency dielectric constant, *ω*—angular frequency, *τ*—dielectric relaxation time constant, and α—exponent parameter describing different spectral shapes.

Readouts of moisture in porous media that utilize the Time Domain Reflectometry method are only based on time-dependence analysis and may be subject to errors. Most calibration methods relate the real part of the complex permittivity to the material moisture. Calibration models used in TDR are divided into physical and empirical ones. One of the main benefits of physical models is their ability to operate independently of calibration tests. The disadvantages include a general mathematical description with many formal abstractive coefficients that require assumptions or are difficult to determine. On the other hand, empirical models require calibration but are easier to apply in practice. Among the empirical models, universal and individual models can be distinguished [45]. Universal models were derived on the basis of research in many different media that differ in structure, porosity, bulk density or chemical composition. Individual models concern a given material or sensor, or are related to a given research procedure [46]. Among the empirical models used for TDR sensor calibration, the Topp model is the most frequently reported for practical moisture assessment of the medium [24]. It is defined by the following equation:

(5) Θ = 0.0000043ε^3^ − 0.00055ε^2^ + 0.0292ε − 0.053

where: Θ—volumetric water content in the examined porous medium [cm^3^/cm^3^] and ε—real part of the complex apparent permittivity of the material determined by TDR [-]. The universal nature of this model does not preclude significant measurement inaccuracies, which have been documented by numerous researchers. [24]. Paper [47] showed that the range 0.05–0.15 cm^3^/cm^3^ describes the possible uncertainty of measurement. There are also models that, in addition to electrical permittivity, take into account parameters such as the density of the tested materials or even temperature, which is intended to improve measurement accuracy [48]. Determining the value of the apparent permittivity allows for the assessment of the moisture content of a material by using theoretical or physical models available in the literature [35] or individual calibration based on experimental tests [27].

Many of the models discussed in the previous paragraph are based on second-order or higher-order polynomial equations. These models take into consideration only the real component of the complex apparent permittivity measured over time. To account for signal attenuation associated with ionic conductivity, moisture content should be evaluated by considering both the signal propagation time and its attenuation. Multiple linear regression (MLR) can be applied for this purpose. MLR is a statistical method widely used for data analysis and modeling [49]. Multiple linear regression extends the simple linear regression model by considering the relationship between a dependent variable and more than one independent variable [50]. While simple linear regression examines the effect of a single independent variable on the dependent variable, MLR allows the simultaneous assessment of multiple predictors [51]. This approach makes it possible to determine how several factors jointly affect the outcome of a given phenomenon or process [52]. The general form of the multiple linear regression model is expressed as follows [53]:

(6) Y = β_0_ + β_1_X_1_ + β_2_X_2_ + … + β_p_X_p_ + e

where: Y—the dependent variable; β_0_—y-intercept; β_1_, β_2_—the regression coefficients, which describe the contribution of each independent variable to the dependent variable; X_1_, X_2_—the independent variables; and e—the error term accounting for the residual variation that remains unexplained by the model. This model can take various forms [54]. Multiple regression equations are often described using polynomials; in this work, second-degree polynomials are used.

After performing MLR analysis, an important step is to evaluate and interpret the obtained results. During the interpretation of the regression model, it is necessary to take the following aspects into account [55]: (1) Regression coefficients, also referred to as β coefficients, indicate the effect of each independent variable on the dependent variable. Positive coefficients indicate a positive relationship, whereas negative coefficients indicate a negative relationship. The larger the absolute value of the coefficient, the stronger the effect of the corresponding independent variable. (2) The adjusted coefficient of determination R^2^_adj_: this coefficient indicates the proportion of the variance in the dependent variable that is explained by the independent variables included in the model. Values closer to 100% indicate that the regression model provides a better description of the observed dependent variable. (3) The residual standard error (RSE): this parameter measures the magnitude of the residual variation, i.e., the differences between the observed and predicted values. A lower RSE indicates a better fit of the regression model to the data, whereas a higher RSE indicates a poorer fit. (4) The root mean square error (RMSE): this parameter measures the standard deviation of the residuals and reflects the magnitude of the errors made by the model when predicting quantitative data. RMSE indicates how closely the observed values are distributed around the fitted regression model. The RMSE value is always non-negative, and lower values indicate better model performance.

The aim of this work was to improve the measurement quality of the Time Domain Reflectometry noninvasive surface sensors by modifying the calibration formulas, which take into account the attenuation of the electromagnetic pulse caused by material moisture and the presence of salt ions, and to develop calibration formulas in the form of multiple linear regression equations.

## 2. Materials and Methods

### 2.1. Materials

The measurements were conducted using TDR equipment [ETest, Lublin, Poland] that consisted of a typical TDR multimeter and surface TDR sensors specifically developed for measuring moisture content in hard porous media, mainly construction materials, and which were described previously in the following articles [26,27]. Other necessary pieces of equipment included a laboratory oven [Chemland, Stargard, Poland], laboratory scales WLC 20/A2 [RADWAG, Radom, Poland] and Merck testers to control the salinity of the samples.

#### 2.1.1. TDR Meter Description

Measurements were performed using a TDR multimeter releasing a needle peak signal with a rise time of 300 ps, manufactured by ETest [Lublin, Poland] [27]. The signal generated by the TDR system was transmitted to the sensor through a coaxial cable. Upon reaching characteristic locations along the propagation path, the signal underwent reflections, with the principal reflections originating at the sensor’s beginning and end. These reflections provided reference points for determining the propagation time. The time interval between the reflected signals was recorded by the TDR meter and subsequently converted, either automatically or manually, into apparent permittivity, which is related to the moisture content of the investigated material.

#### 2.1.2. Noninvasive TDR Sensors

The experiment used two surface TDR sensors designed at the Lublin University of Technology. These sensors were previously presented in an article by Suchorab et al. [37].

#### 2.1.3. Wide Noninvasive Sensor

**Figure 1:**
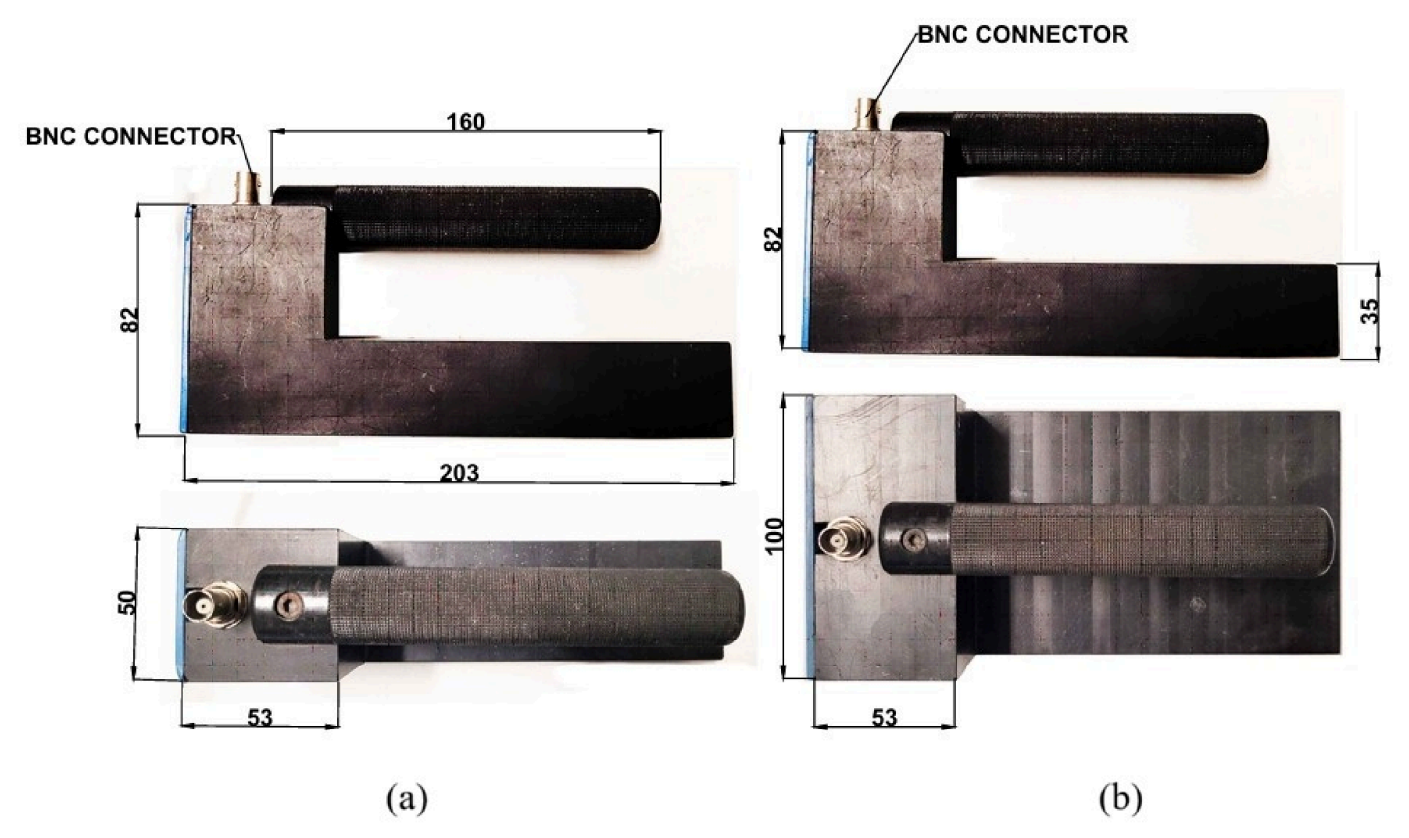
(**a**) Narrow Sensor; (**b**) Wide Sensor.

A wide, noninvasive surface sensor, called Wide Sensor in this article, presented in Figure 1b, is manufactured from black polyoxymethylene with an apparent permittivity of 3.8. The sensor is 200 mm long and 50 mm wide. The measuring rods have a flat cross-section of 2 × 10 mm and are made of brass. Signal transfer between the sensor and the TDR multimeter is achieved via an angled BNC connector attached to a printed circuit board connecting the measuring rods to the connector.

#### 2.1.4. Narrow Noninvasive Sensor

The narrow, noninvasive surface sensor, called the Narrow Sensor, presented in Figure 1a, has a similar configuration. It is made of the same material as the Wide Sensor. Like the Wide Sensor, it is 200 mm long and 100 mm wide, and the sensor waveguides are made of a 2 × 10 mm brass flat bar.

#### 2.1.5. Sample Description

Ceramic brick (red brick), clinker brick, and silicate brick were used for the tests as the building materials. All samples had regular brick dimensions of 25 cm × 12.5 cm × 6 cm. Table 1 presents averaged basic physical parameters of all samples of the used materials.

### 2.2. Methods

The samples were prepared according to the following procedure. Sodium chloride (NaCl) and distilled water were used to prepare the necessary salt solutions. In order to achieve salt concentrations corresponding to the low, medium and high states, test solutions with the following mass concentrations were prepared: 0.45%, 1.5%, 5%. The samples were moistened to saturation in sodium chloride solutions, with each sample in a solution of a different concentration. Full saturation was achieved using the weighing method, and no weight gain was observed during subsequent measurements. Salt concentration tests in test samples were performed using MERCK tests. For the test, 15 samples of each material were used (5 samples of each level of salinity). Next, the bricks were dried sequentially until the assumed moisture levels were reached depending on material type. During drying, the prepared samples were sequentially verified for moisture. For the purposes of conducting the experiment and developing calibration models for red ceramic bricks, the following control levels of volumetric water content were established: 0, 0.0161, 0.0322, 0.0483, 0.0644, 0.0805, 0.0966, 0.1127, 0.1288, 0.1449, 0.161, 0.1771, 0.1932, 0.2093, and 0.2254 [cm^3^/cm^3^], which corresponded to the mass moisture of the material in [%]. For clinker brick, the following volumetric water content values were established: 0, 0.0219, 0.0438, 0.0657, 0.0876, and 0.1095 [cm^3^/cm^3^], and in the case of silicate brick samples, the analysis was conducted for the following moisture contents: 0, 0.0362, 0.1086, 0.1448, 0.181, and 0.2353 [cm^3^/cm^3^].

At each moisture level, the samples were tested with the TDR multimeter, and waveforms were collected, which were subsequently utilized in further data processing. As a result, relationships were obtained between the electrical data of the materials and material moisture. Three approaches were used: (1) deriving a general calibration model independent of salinity levels θ(ε)_gen_, (2) deriving the individual calibration model dependent on salinity level θ(ε)_ind_, (3) deriving the general calibration model that includes both moisture and salinity θ(ε,V) using MLR (multiple linear regression).

## 3. Results

**Figure 2:**
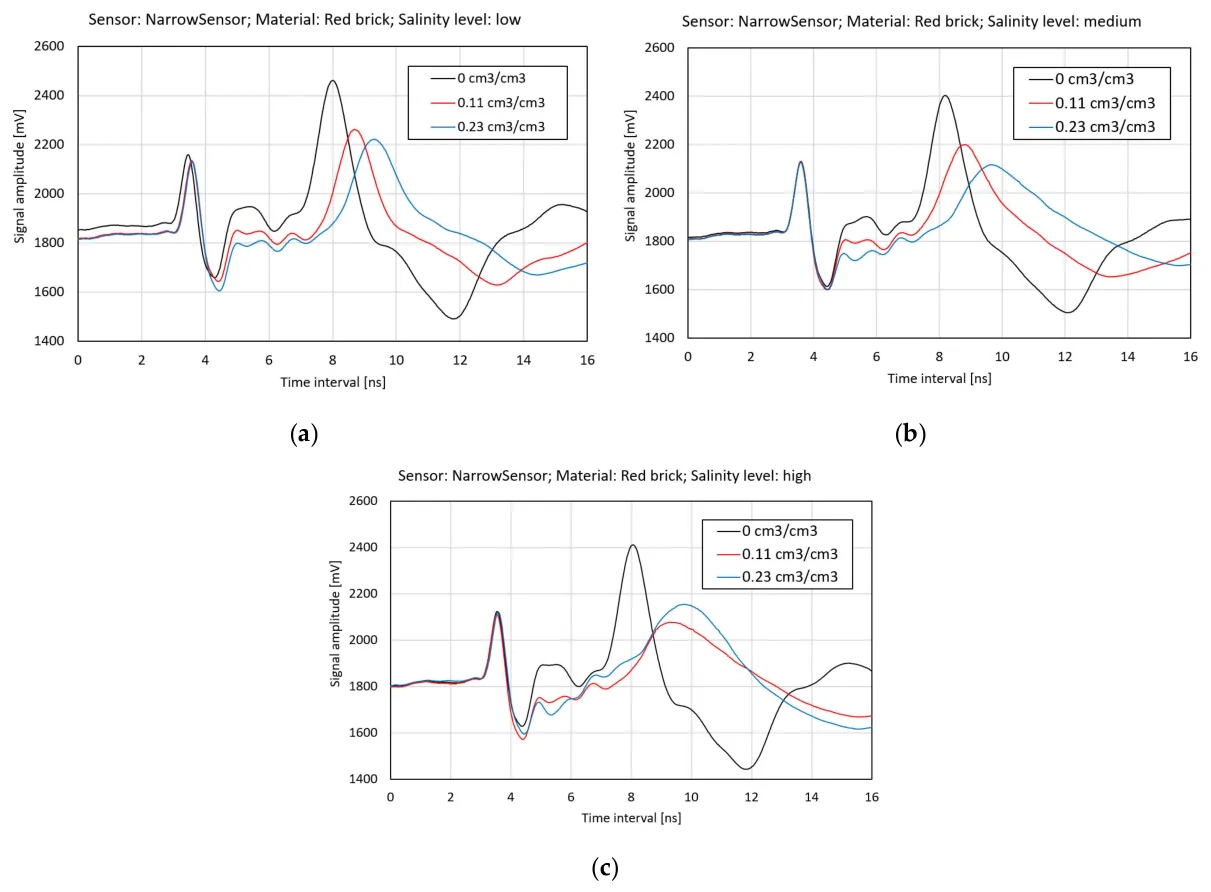
Waveforms collected using a Narrow Sensor from red brick samples for different concentrations of chlorides ((**a**)—low, (**b**)—medium, (**c**)—high).

**Figure 3:**
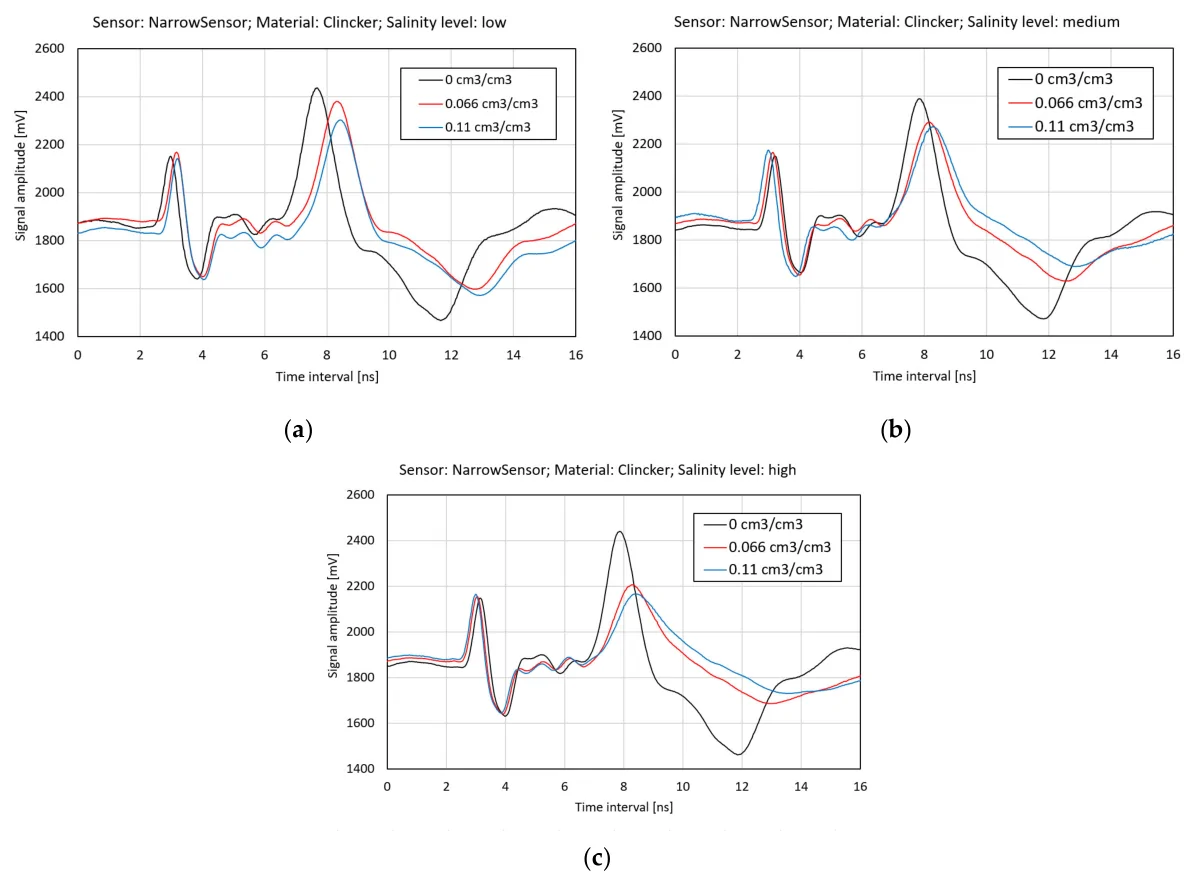
Waveforms collected using a Narrow Sensor from clinker samples for different concentrations of chlorides ((**a**)—low, (**b**)—medium, (**c**)—high).

**Figure 4:**
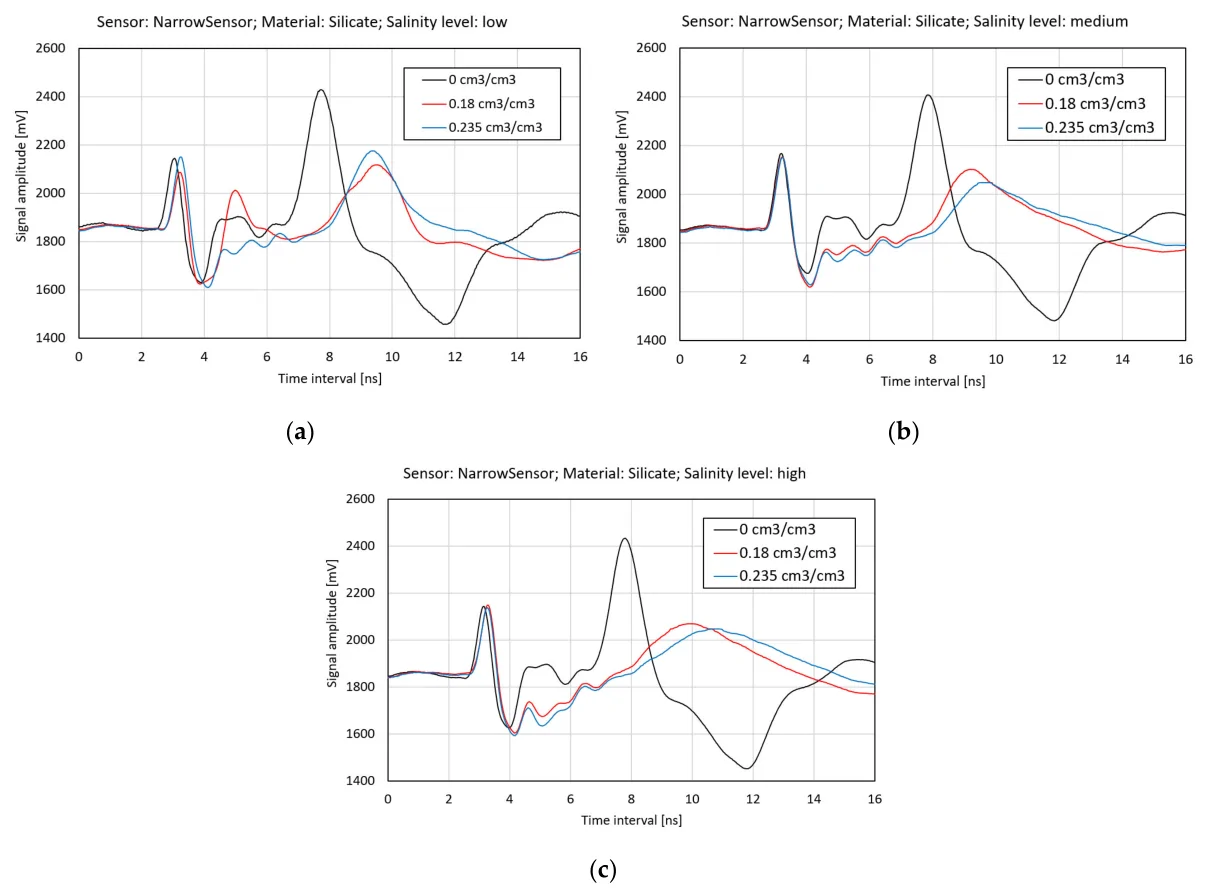
Waveforms collected using a Narrow Sensor from silicate samples for different concentrations of chlorides ((**a**)—low, (**b**)—medium, (**c**)—high).

The results of the experiment consisted of the set of raw data obtained from the TDR multimeter, which were combined with the moisture and salinity of samples that were prepared under laboratory conditions. The diagrams below present exemplary waveforms achieved for the samples that differ in moisture and salinity using a Narrow TDR Sensor (Narrow Sensor). Figure 2 shows data achieved for red brick, Figure 3 for clinker and Figure 4 for silicate brick. To show the wide possible variability of data, different moisture states were selected. In the case of red brick (Figure 2), the following moisture values were selected: 0 cm^3^/cm^3^ (dry material), 0.11 cm^3^/cm^3^ (intermediate wet material) and 0.23 cm^3^/cm^3^ (saturated material). For clinker (Figure 3), waveforms of the following moistures were selected: 0 cm^3^/cm^3^, 0.066 cm^3^/cm^3^ and 0.11 cm^3^/cm^3^. In the case of the silicate (Figure 4), the selected values were 0 cm^3^/cm^3^, 0.18 cm^3^/cm^3^ and 0.235 cm^3^/cm^3^, respectively.

The diagrams indicate a clear shift in the position of the second positive peak toward longer propagation times as the moisture content increases. This behavior corresponds to a longer signal travel time and, consequently, to higher values of apparent permittivity. At the same time, an increase in salinity results in a reduction in the amplitude of the second peak, accompanied by lower recorded voltage values. This effect can be attributed to the contribution of the imaginary component of the complex permittivity. This difference is significantly visible in Figure 1 for low and medium concentrations of chlorides. In the case of high concentrations of chlorides, there is a visible time shift between data readings, but the phenomenon of signal attenuation is not that clear. For dry and 0.11 cm^3^/cm^3^ wet materials, this rule works according to expectations, but in the case of saturated material, the voltage increases. Similar observations were made when other materials were used in the research.

**Figure 5:**
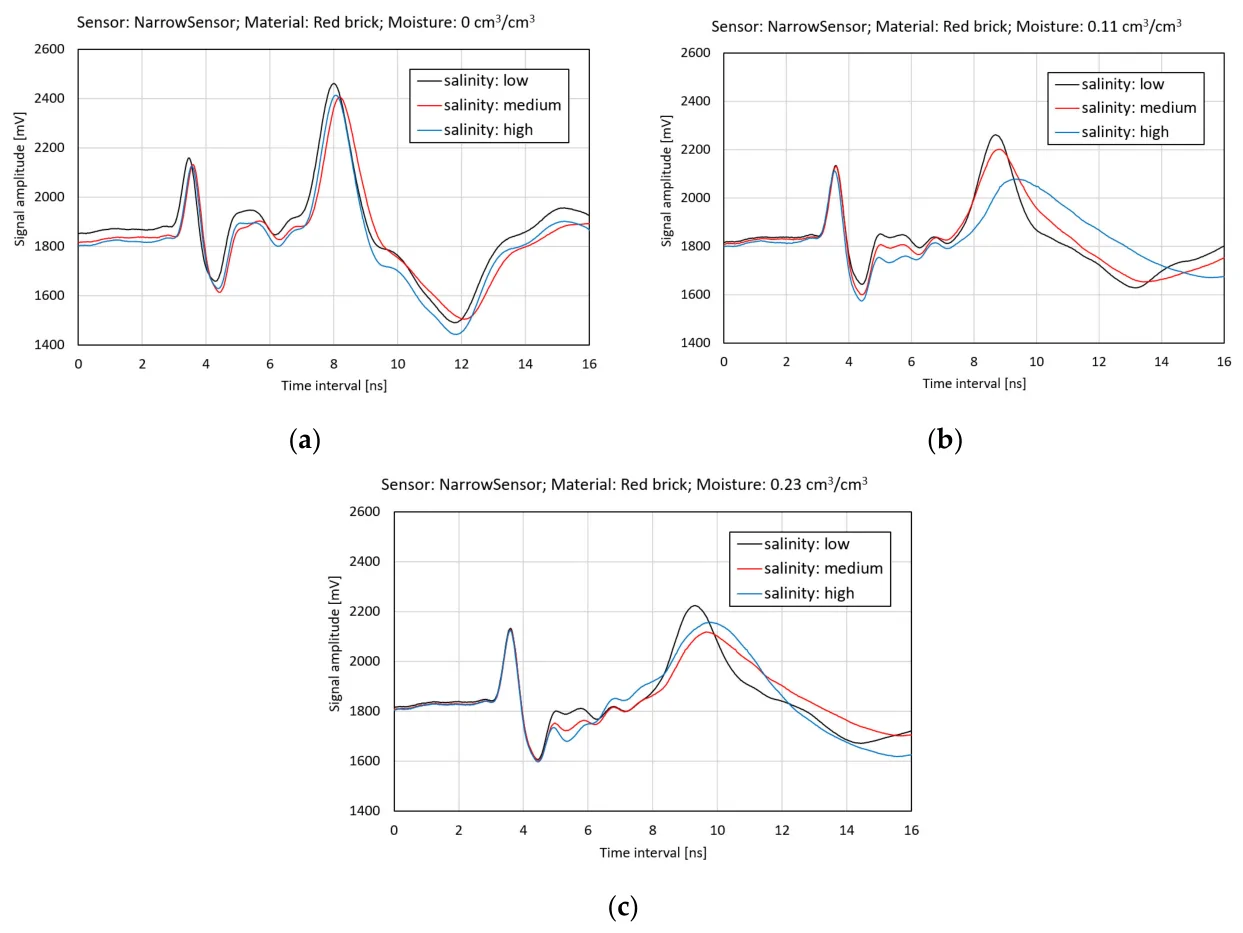
Waveforms collected using a Narrow Sensor from red brick samples for different moisture values ((**a**)—0 cm^3^/cm^3^, (**b**)—0.11 cm^3^/cm^3^, (**c**)—0.23 cm^3^/cm^3^).

**Figure 6:**
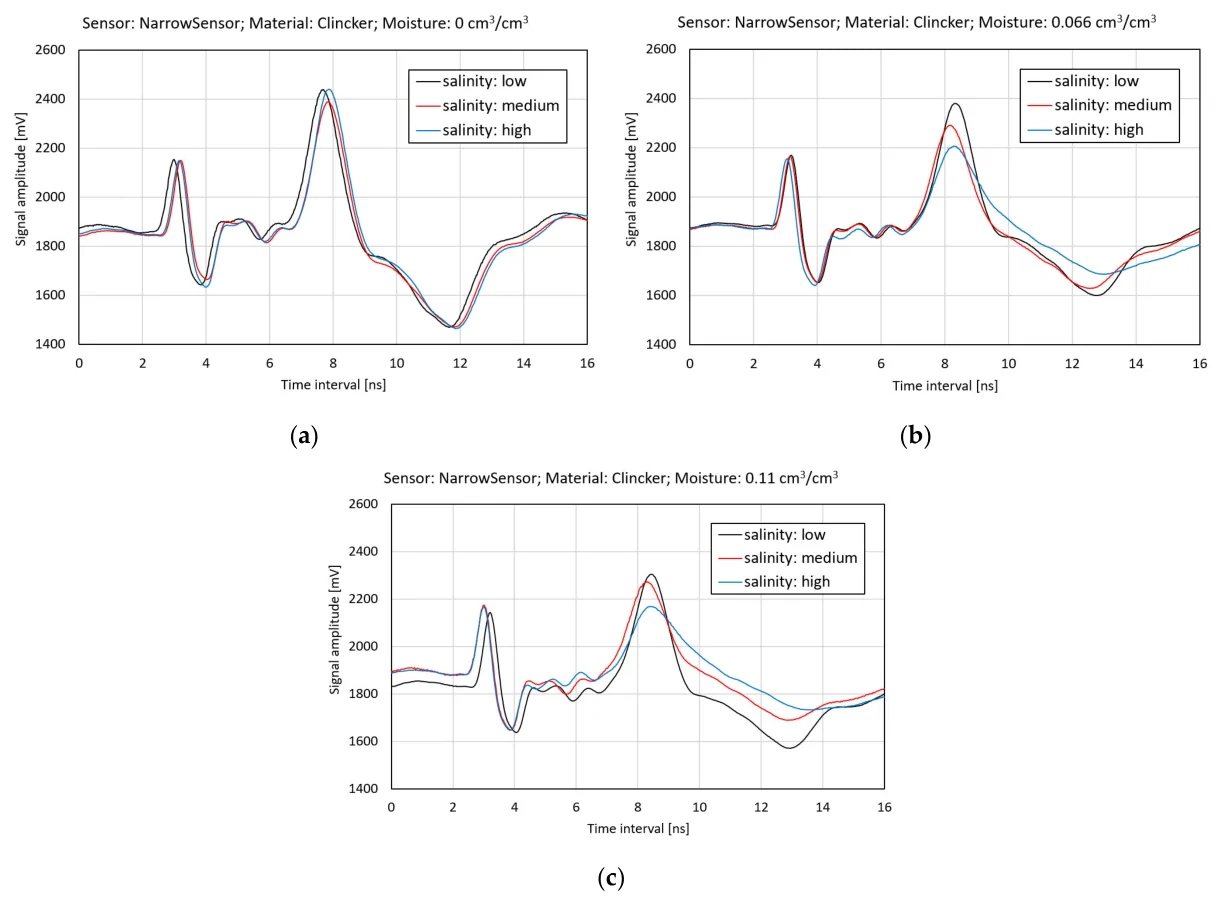
Waveforms collected using a Narrow Sensor from clinker samples for different moisture values ((**a**)—0 cm^3^/cm^3^, (**b**)—0.11 cm^3^/cm^3^, (**c**)—0.23 cm^3^/cm^3^).

**Figure 7:**
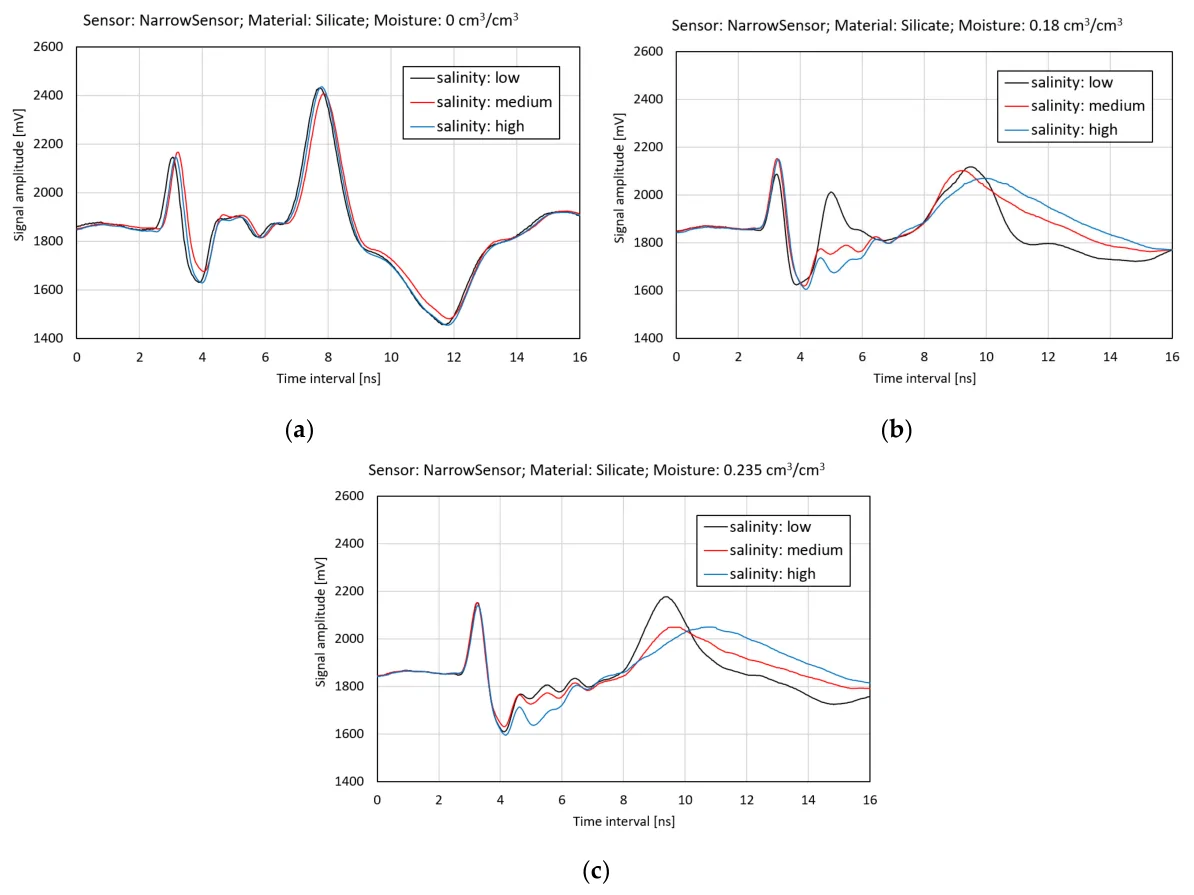
Waveforms collected using a Narrow Sensor from silicate samples for different moisture values ((**a**)—0 cm^3^/cm^3^, (**b**)—0.11 cm^3^/cm^3^, (**c**)—0.23 cm^3^/cm^3^).

Interestingly, the same data can be presented for constant moisture values and different salinity values. Figure 5, Figure 6 and Figure 7 show these dependences for all tested materials.

These diagrams reveal the influence of salinity concentration on signal attenuation. In the case of dry material (0 cm^3^/cm^3^), this influence is not significant; only small differences are noted. The most interesting are the diagrams for average and saturated states, where a clear relation between material salinity and signal attenuation is revealed. Here, it is clearly noticeable that the time of propagation increases with the decrease in signal amplitude, even in the case of the samples with constant moisture values.

## 4. Discussion

### 4.1. Evaluation of Apparent Permittivity

Having collected the raw data from the TDR multimeter, it was possible to estimate the apparent permittivity values with Formula (5) for each sensor type, material, moisture and salinity level. To evaluate apparent permittivity values, the raw TDR signal had to be interpreted by estimating the time marker positions. Those positions were read from each diagram, and the time span between the negative peak and the positive peak was measured. From this measured value, the “dead time” value was subtracted, which was estimated for each sensor and multimeter in the calibration procedure. The general formula to evaluate signal propagation time through the sensing elements of the TDR sensors was the following:

(7) t_p_ = t_meas_ − t_dead_ [ns]

where: t_p_—signal propagation time through the measuring elements, [ns]; t_meas_—time measured between two peaks on the waveform (negative on resistor and positive on sensor termination), [ns]; and t_dea(d)—_time of signal propagation along the printed circuit between the resistor and the beginning of the sensing element (constant for a particular sensor specimen and multimeter, established in calibration), [ns]. By estimating the t_p_ time, it was possible to evaluate the real value of the complex apparent permittivity; it was estimated using Formula (1), where the length of the sensor equals 20 cm.

Additionally, for further data analysis, voltage readouts from the second (positive peaks) were evaluated, which were later utilized to enrich model moisture evaluation.

### 4.2. Dependence Between Apparent Permittivity and Volumetric Water Content—θ(ε)

Having determined the values of electrical permittivity for all moisture states of the samples, calibration models, which are polynomial-type equations, were established for a specific number of repetitions. The assumed model of the calibration formulas is a second-degree polynomial. The input data to the model were material moisture determined by direct gravimetric tests and the average value of apparent permittivity obtained by reflectometry measurements:

(8) θ(ε) = β_0_ + β_1_ ⋅ ε + β_2_ ⋅ ε^2^ [cm^3^/cm^3^]

where θ(ε)—mass moisture of the material determined using a polynomial model.

The relationship between material moisture content and the apparent permittivity measured by the TDR equipment can be determined using two different approaches—the first and general approach is to find the regression formula θ(ε)_gen_ independently of salinity, which is the most common approach in TDR measurement. The second, more detailed approach is to derive the formulas θ(ε)_ind_ for different levels of salinity, which is more difficult because it requires recognition of salinity concentration. In this subchapter, both approaches are presented.

**Figure 8:**
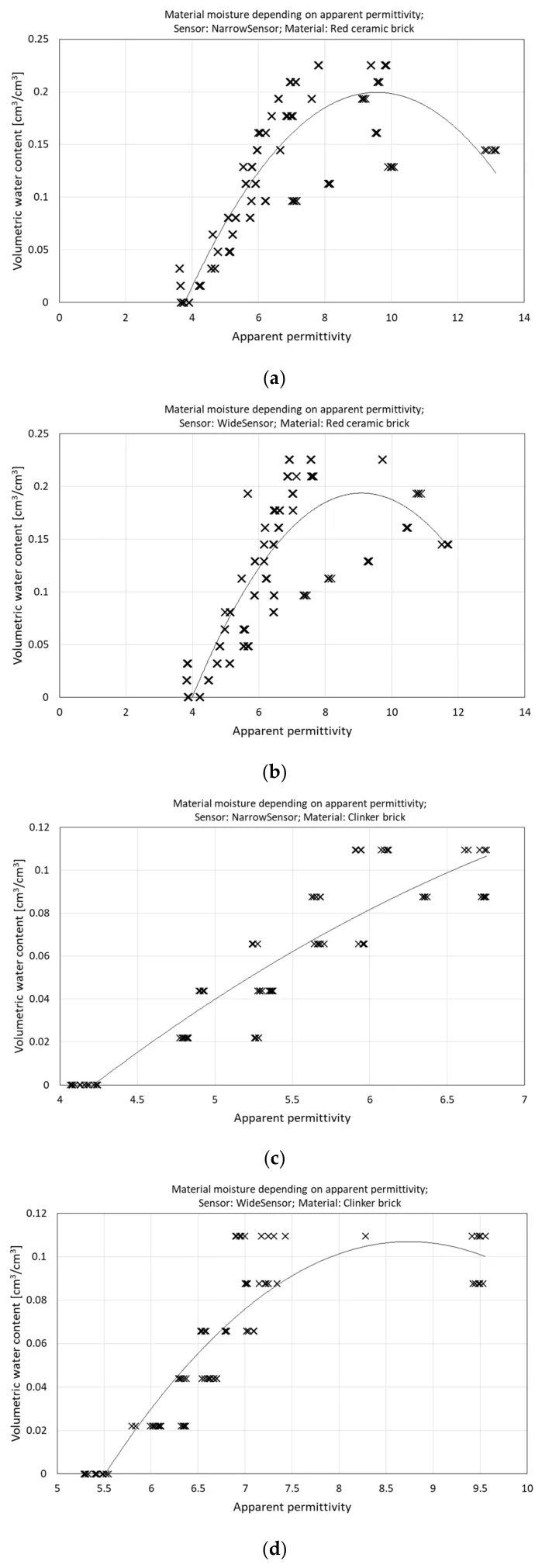

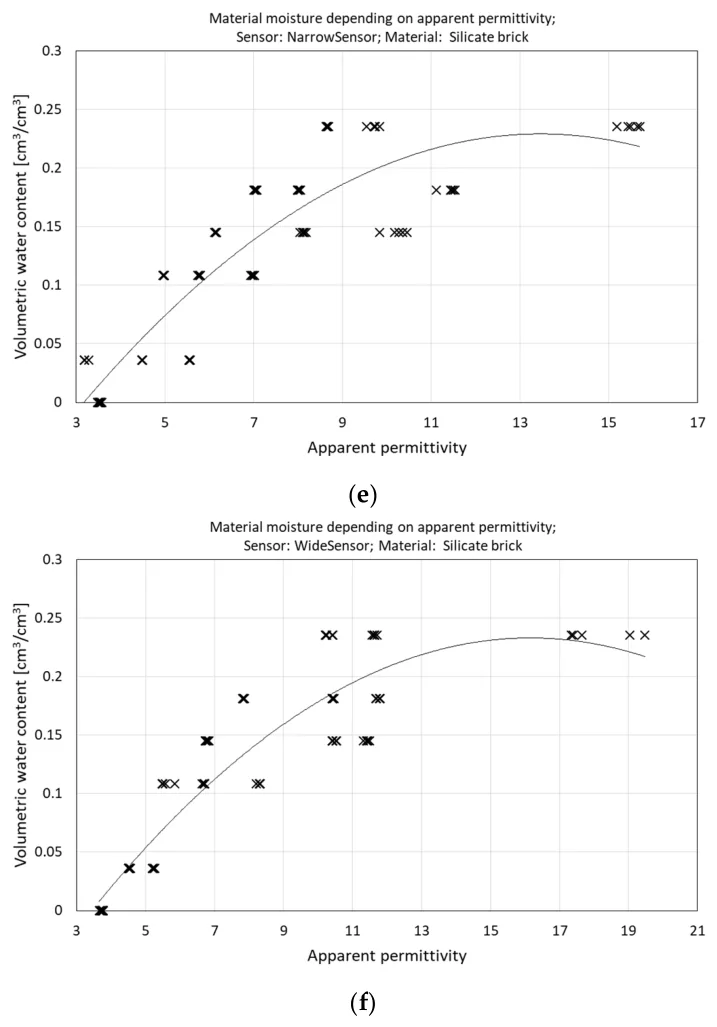
The generalized θ(ε)_gen_ relationships describing the dependence of TDR-based apparent permittivity on material moisture for all investigated materials measured and two types of sensors used, independently of the salinity level ((**a**)—red brick, Narrow Sensor, (**b**)—red brick, Wide Sensor, (**c**)—clicker brick, Narrow Sensor, (**d**)—clicker brick, Wide Sensor, (**e**)—silicate brick, Narrow Sensor, (**f**)—silicate brick, Wide Sensor).

First, the general dependences θ(ε)_gen_ between apparent permittivity and volumetric water content were derived for all measurements done for each material and sensor type independently of salinity levels. These dependences are visible in Figure 8.

**Figure 9:**
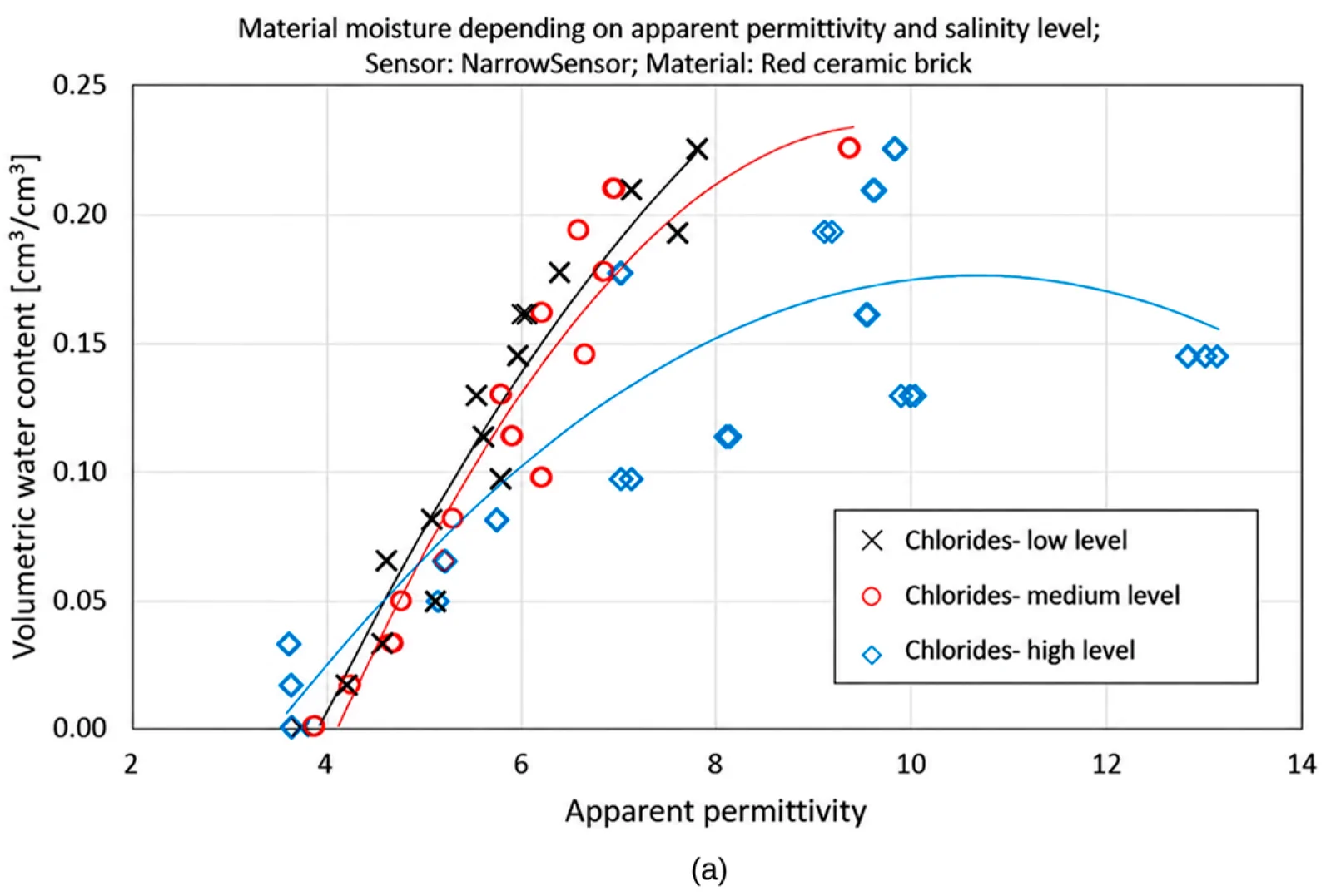

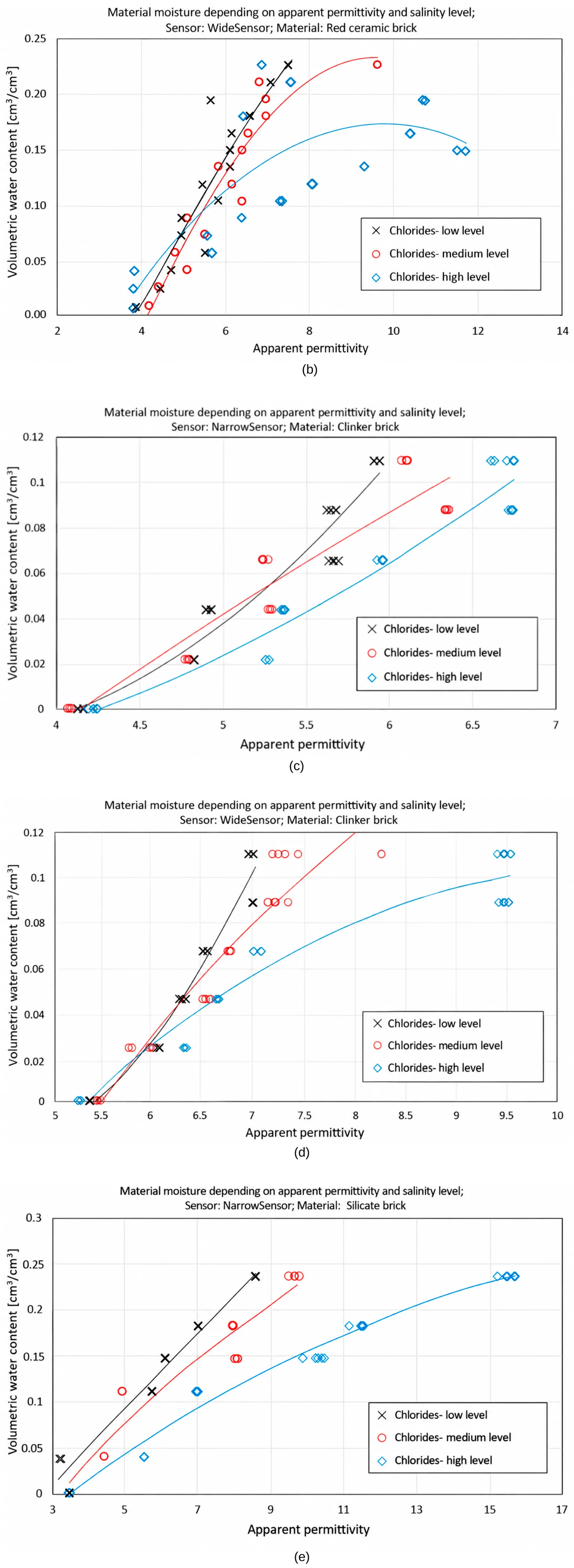

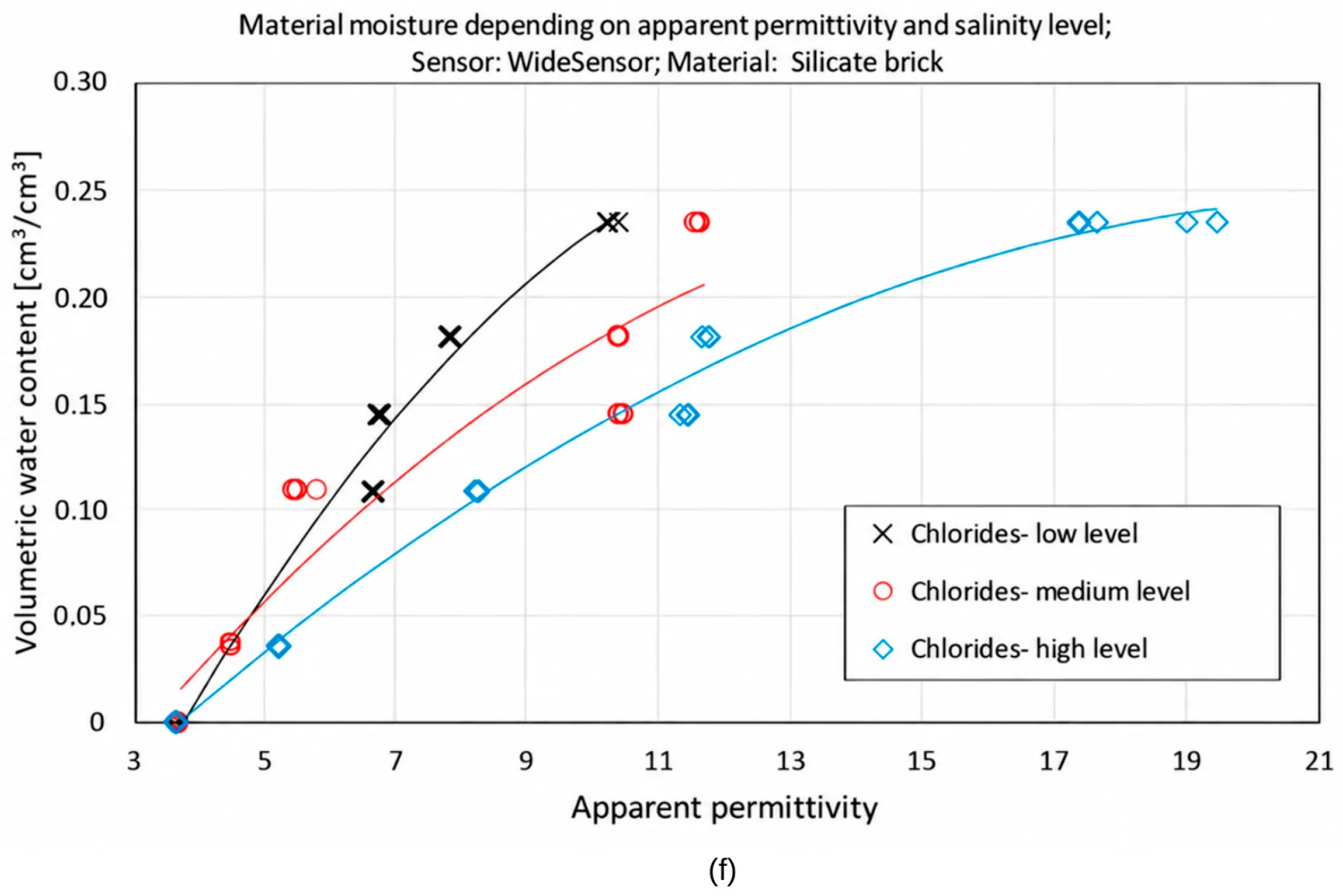
The generalized θ(ε)_ind_ relationships describing the dependence of TDR-based apparent permittivity on material moisture for three different levels of salinity, two types of sensors used and three types of bricks ((**a**)—red brick, Narrow Sensor, (**b**)—red brick, Wide Sensor, (**c**)—clinker brick, Narrow Sensor, (**d**)—clinker brick, Wide Sensor, (**e**)—silicate brick, Narrow Sensor, (**f**)—silicate brick, Wide Sensor).

A more detailed presentation of the same dependences is presented in the diagrams in Figure 9, where the relation θ(ε)_ind_ between apparent permittivity and material moisture for three different levels of salinity is revealed. The colored lines represent polynomial fits to the corresponding data: black for low chloride levels, red for medium levels, and blue for high levels.

With the results presented in Figure 8 and Figure 9, regression formulas that show the relations between apparent permittivity evaluated by the TDR and volumetric water content of the materials were derived. The formulas are second-grade polynomial regression functions based on Formula (5). Six formulas, which are presented in Table 2, describe the dependence θ(ε)_gen_ between apparent permittivity and material moisture independently of salinity level; on the other hand, the formulas presented in Table 3 are derived for each salinity level separately, θ(ε)_ind_.

In Table 2 and Table 3, all derived calibration formulas for moisture evaluation are presented. Moreover, Table 2 and Table 3 show the coefficients of determination (R^2^) values, Residual Standard Error (RSE) and Root Mean Squared Error (RMSE) values. It can be seen that the R^2^ coefficient values are higher for formulas θ(ε)_ind_ derived for each salinity level individually, and RSE and RMSE values are lower. This means that when considering the presence of salt ions, the regression formulas are better in phenomenon description and provide better accuracy of measurement. In the case of the general formulas θ(ε)_gen_, R^2^ coefficients take the values between 0.704 and 0.859, while in the case of the formulas derived for particular salinity levels, those coefficients vary from 0.627 to 0.993, which means that from 70% to 86% of the variability of the sample of the dependent variable (moisture) is explained by the variability of the model (variability of ε) in the case of general formulas, while in the case of the detailed formulas, this value reaches 99% (silicate brick, Narrow Sensor). For general θ(ε)_gen_ formulas, RSE varies from 0.015 cm^3^/cm^3^ for clinker brick to 0.031 for red and silicate brick, while for the detailed formulas θ(ε)_ind_, it is between 0.007 and 0.043 cm^3^/cm^3^ depending on material and salinity concentration. Also, the smallest RMSE values (for clinker brick) reach 0.015 cm^3^/cm^3^ for the general formulas θ(ε)_gen_ and are significantly higher compared to those for the similar material and sensor in the case of the individual formulas θ(ε)_ind_ derived for different levels of salinity, where values between 0.009 and 0.012 cm^3^/cm^3^ are achieved.

Additionally, it ought to be mentioned that the best fit of data in almost all cases of materials and sensor types is represented for low levels of chloride concentration. Also, the values of apparent permittivity ranges are smaller compared to other levels of chlorides. In the case of the higher level of chlorides, data dispersion is higher, and the range of apparent permittivity values is higher.

### 4.3. Signal Attenuation Depending on Moisture and Chloride Level

**Figure 10:**
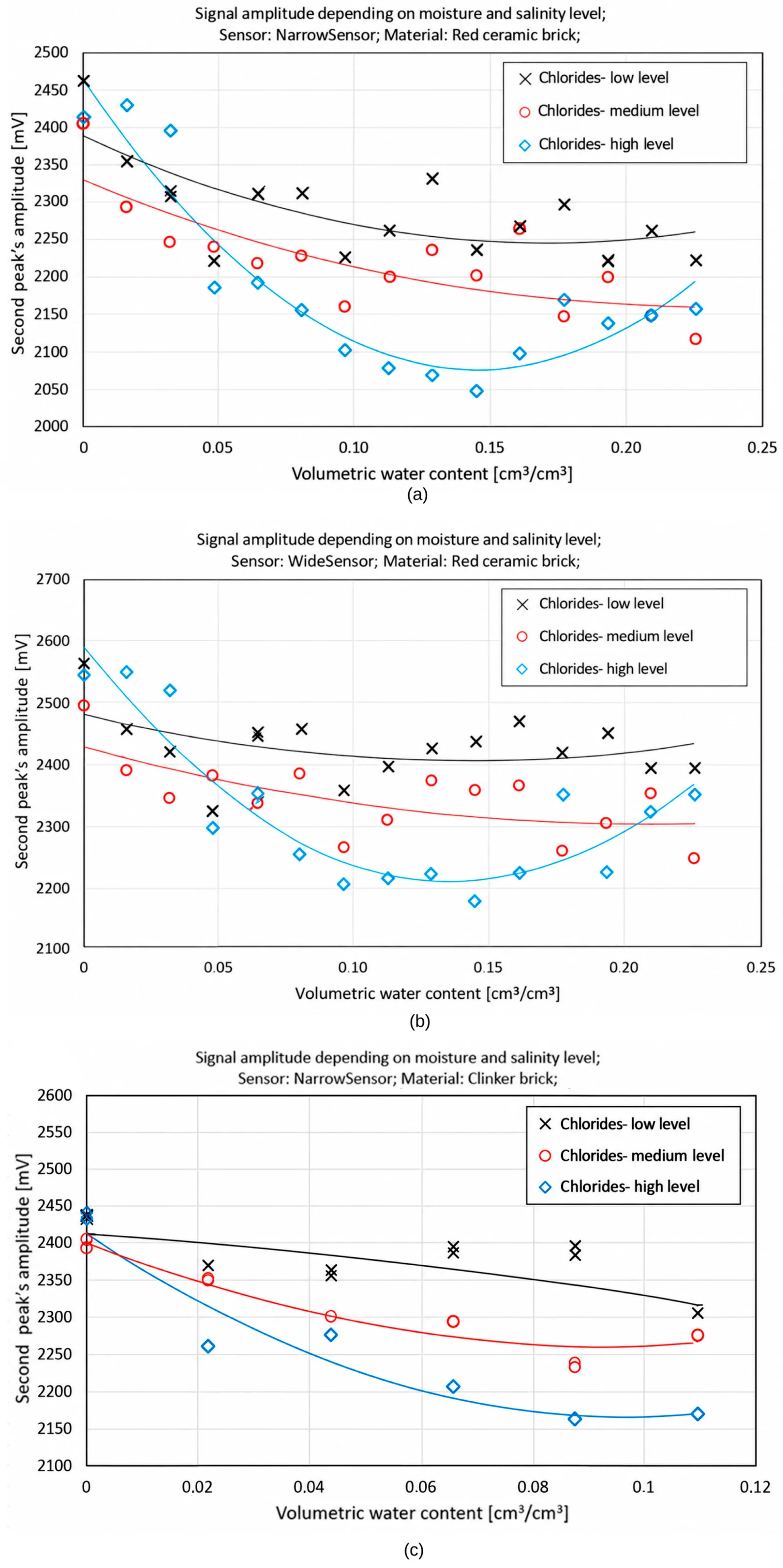

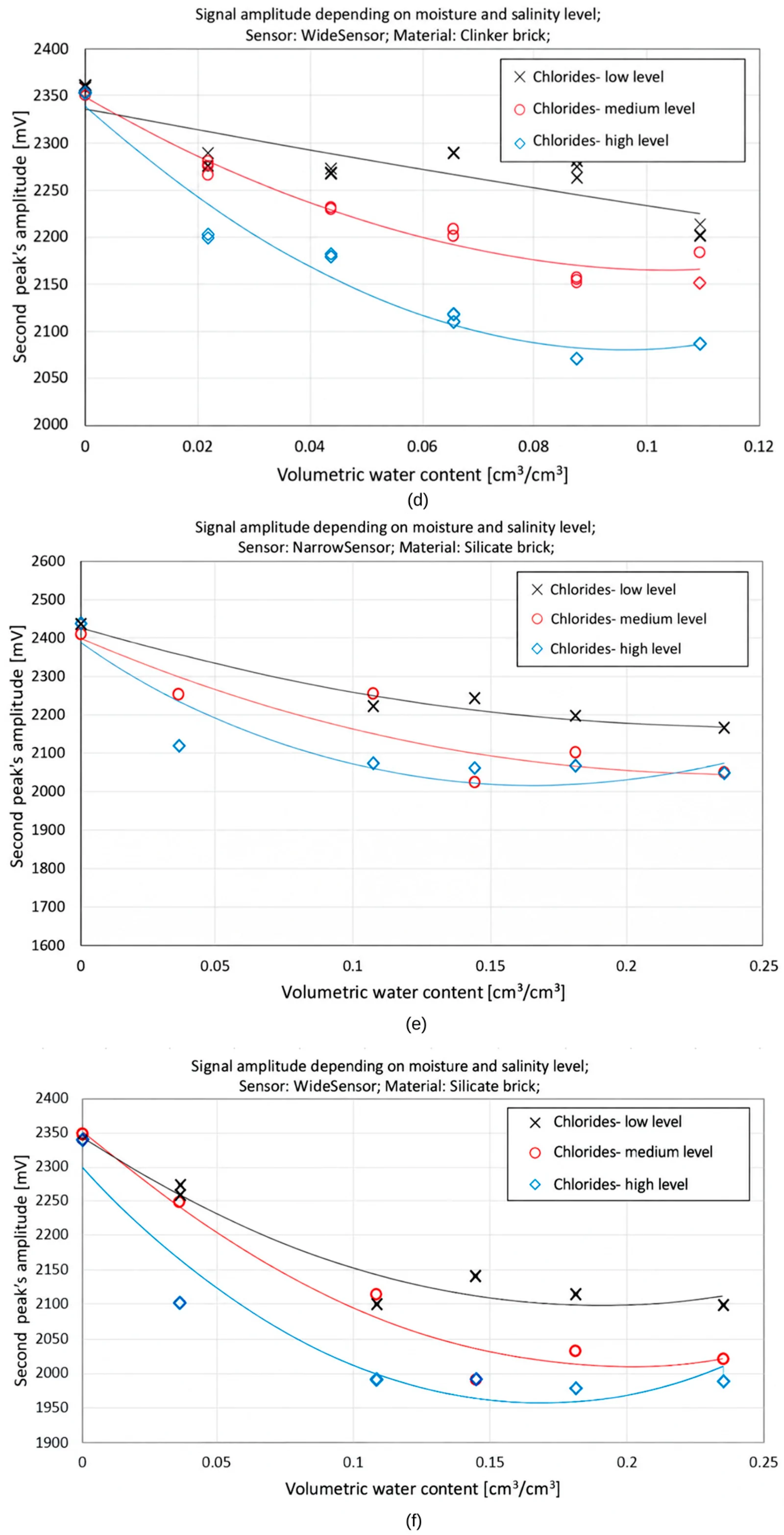
Relationship between the TDR-measured signal voltage at the sensor termination and material moisture at three different salinity levels ((**a**)—red brick, Narrow Sensor, (**b**)—red brick, Wide Sensor, (**c**)—clinker brick, Narrow Sensor, (**d**)—clinker brick, Wide Sensor, (**e**)—silicate brick, Narrow Sensor, (**f**)—silicate brick, Wide Sensor).

Treating permittivity as a complex number, the influence of salinity on signal readout amplitude attenuation ought to be considered. Figure 10 presents the values of voltage read at the second peak of the waveform depending on material moisture and chloride concentration for all types of materials and sensor constructions. The lines represent polynomial fits: black for low-level chlorides, red for medium-level, and blue for high-level.

As can be seen, in the case of a low level of salinity, the attenuation effect is low, and in the case of red ceramic brick, it reaches about 240 mV between dry and saturated material. In the case of medium concentration, the difference is more visible (about 280 mV), and finally, in the case of high concentration of chlorides, the difference is large but more irregular and reaches a maximum of 360 mV in the case of 0.15 cm^3^/cm^3^ wet samples; beyond this, the tendency is irregular, and the attenuation effect is less visible. Similar tendencies are observed for other bricks, but in the case of clinker brick with a high level of chloride concentration, amplitude attenuation is more visible.

**Figure 11:**
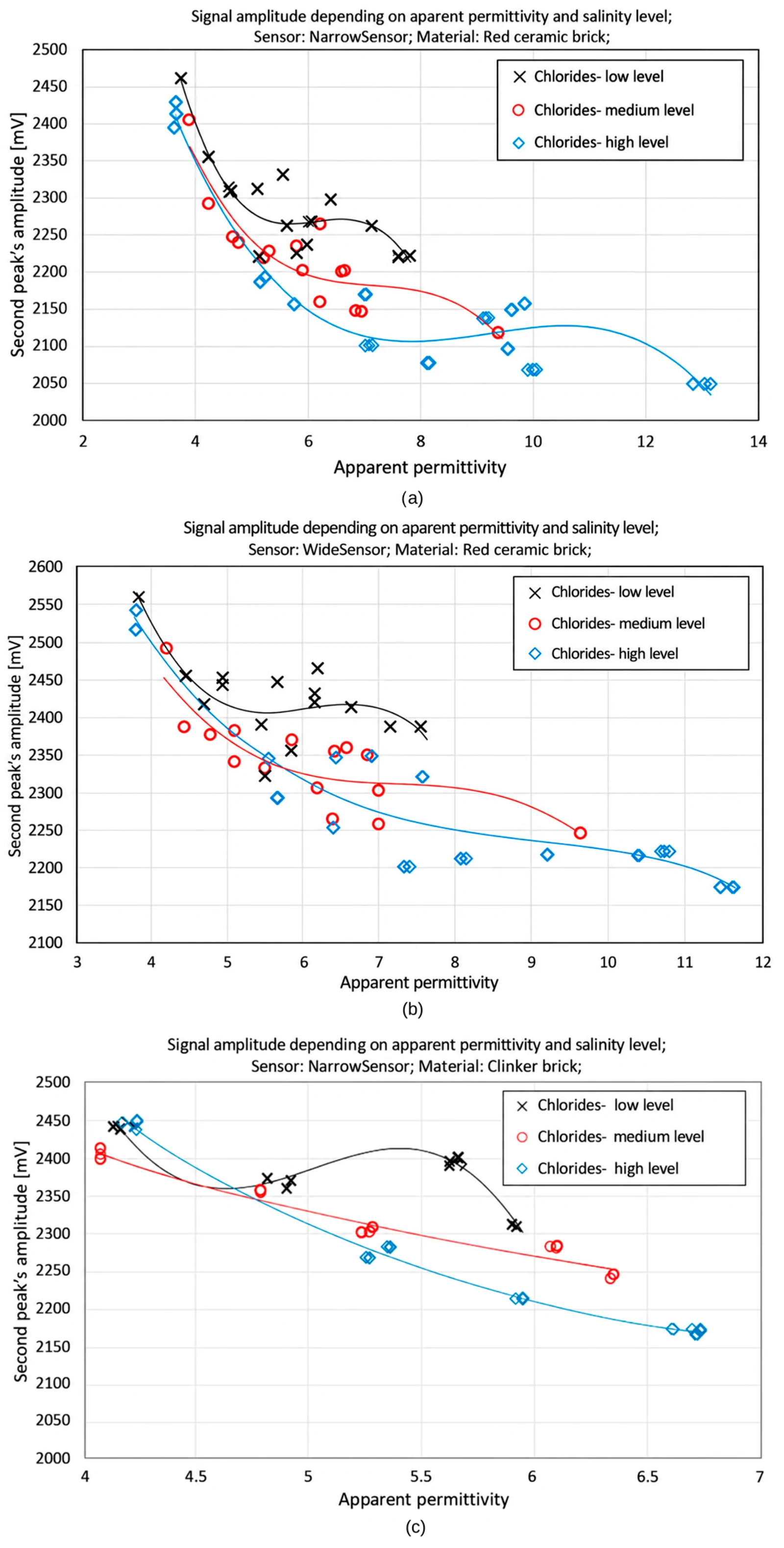

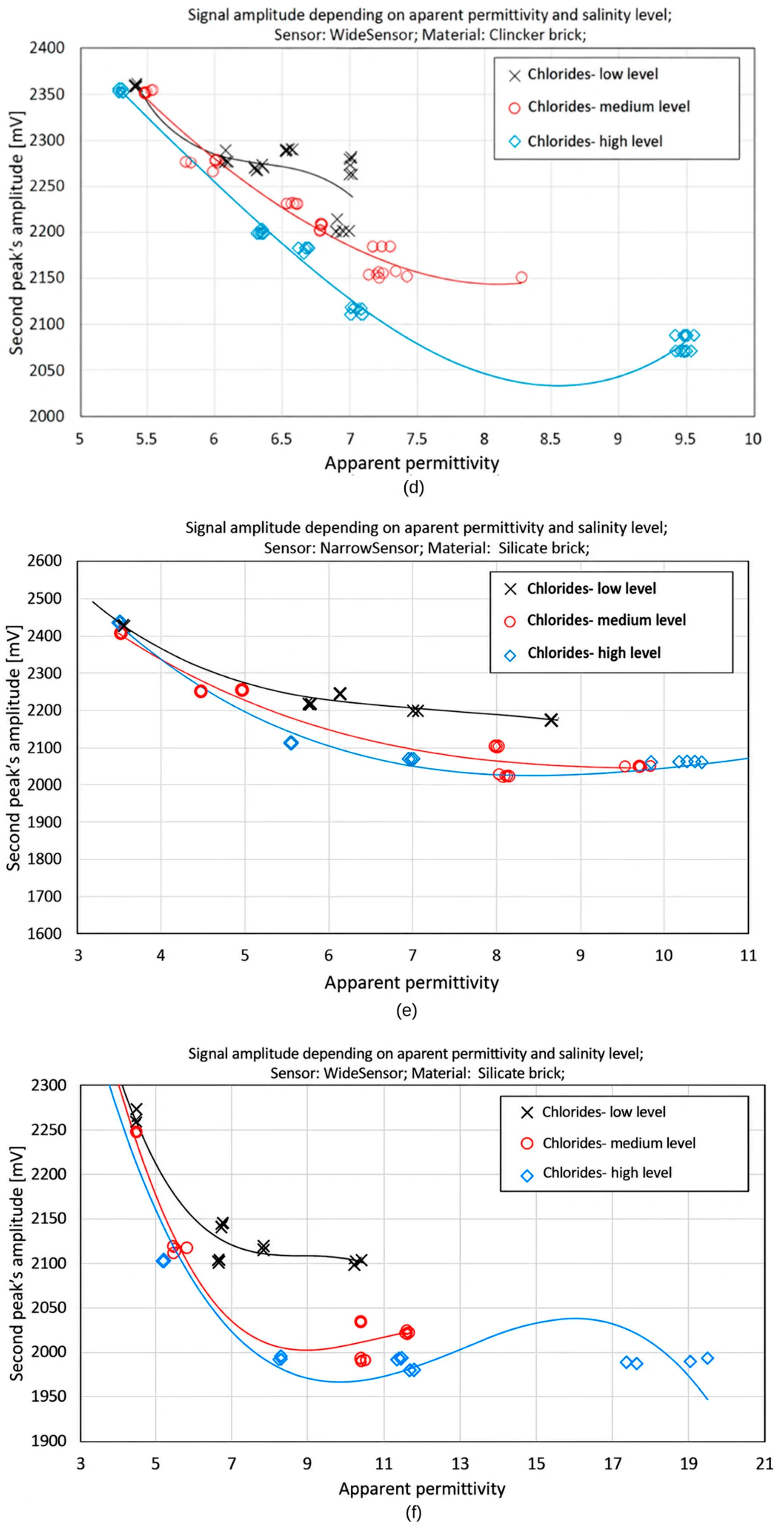
TDR-measured signal voltage at the sensor termination as a function of material moisture for three salinity levels and two sensor types ((**a**)—red brick, Narrow Sensor, (**b**)—red brick, Wide Sensor, (**c**)—clinker brick, Narrow Sensor, (**d**)—clinker brick, Wide Sensor, (**e**)—silicate brick, Narrow Sensor, (**f**)—silicate brick, Wide Sensor).

An interesting dependence can be observed when these two diagrams are combined into one that presents the relationship between apparent permittivity and second peak amplitude. This is presented in Figure 11. The lines represent polynomial fits: black for low-level chlorides, red for medium-level, and blue for high-level.

In this diagram, it is clearly visible that, together with the apparent permittivity increase, signal attenuation is visible. This diagram also partially compensates for the disturbances in the intermediate moisture levels of the material, where the dispersion of apparent permittivity readouts and thus signal attenuation was the greatest. Table 4 presents the formulas derived based on the data in Figure 11 that combine the second peak’s voltage depending on apparent permittivity for each material, salt concentration and sensor type.

The third-degree polynomial functions presented in Table 4 were selected to adequately describe the experimentally observed nonlinear relationship between the voltage amplitude V and the apparent permittivity ε within the investigated measurement ranges. The choice of the third-degree polynomial was based on the observed shape of the experimental V(ε) relationship and the resulting goodness of fit. Importantly, these functions are used to characterize the experimental relationship between the electrical parameters and do not constitute the final moisture calibration equations. For moisture estimation, a second-degree multiple regression model was subsequently adopted, in which ε and V, together with their squared terms, are treated as explanatory variables. The second-degree form was selected to provide sufficient flexibility to account for the nonlinear dependence of moisture on the measured electrical parameters while maintaining a relatively simple and interpretable calibration equation.

### 4.4. Development of Multiple Regression Models—θ(ε,V)

The aim was to derive six regression models describing the relationship between moisture (explained variable) and apparent permittivity and voltage (explanatory variables) for the following materials: ceramic brick, clinker brick and silicate brick. In each case, measurements were made using two types of sensors, namely a Wide Sensor and a Narrow Sensor. To achieve this specific goal of the work, an econometric linear multiple regression model estimated using the least squares method was assumed:

(9) θ(ε,V) = a_0_ + a_1_ε + a_2_ε^2^ + a_3_V + a_4_V^2^ [cm^3^/cm^3^]

where: θ(ε,V)—denotes estimated volumetric water content with the multiple regression model; ε—apparent permittivity; V—voltage; and a_0_, a_1_, a_2_, a_3_, a_4_—structural parameters of the model estimated by the least squares method. The polynomial relationships V(ε) presented in Table 4 should not be interpreted as a direct substitution into Equation (9); rather, they describe the experimentally observed relationship between the two measured electrical parameters. Equation (9) uses both ε and V as explanatory variables in order to retain the information contained in both the signal propagation time and signal attenuation. In order to avoid impossible combinations of electrical permeability and voltage amplitudes, the ranges of possible values that they take have been defined. These were determined through experimental research and are presented in Table 5.

On the basis of the obtained data, six multiple regression models were derived, which are second-degree polynomials of two variables. The equations of these models are presented in Table 6 together with the parameters describing the degree of fit of the models.

The adjusted determination coefficient R^2^_adj_ with values from 0.873 to 0.955 means that from 87% to 96% of the variability of the sample of the dependent variable (moisture) is explained by the variability of the model (variability of ε and V). The RSE value varies from 0.011 cm^3^/cm^3^ to 0.027 cm^3^/cm^3^, while the RMSE value varies from 0.012 cm^3^/cm^3^ to 0.026 cm^3^/cm^3^.

The regression models presented above were then used to determine the volumetric water content of the materials based on the fixed values of apparent permittivity ε and voltage V. On the basis of laboratory measurements, it was assumed that the variable ε ranges from 3.4 to 16.6. The values of the V variable were determined using trend line equations (polynomial equations of the third degree), which were determined for three groups, taking into account the salinity level (low, medium, high); see Table 6 and Table 7.

**Figure 12:**
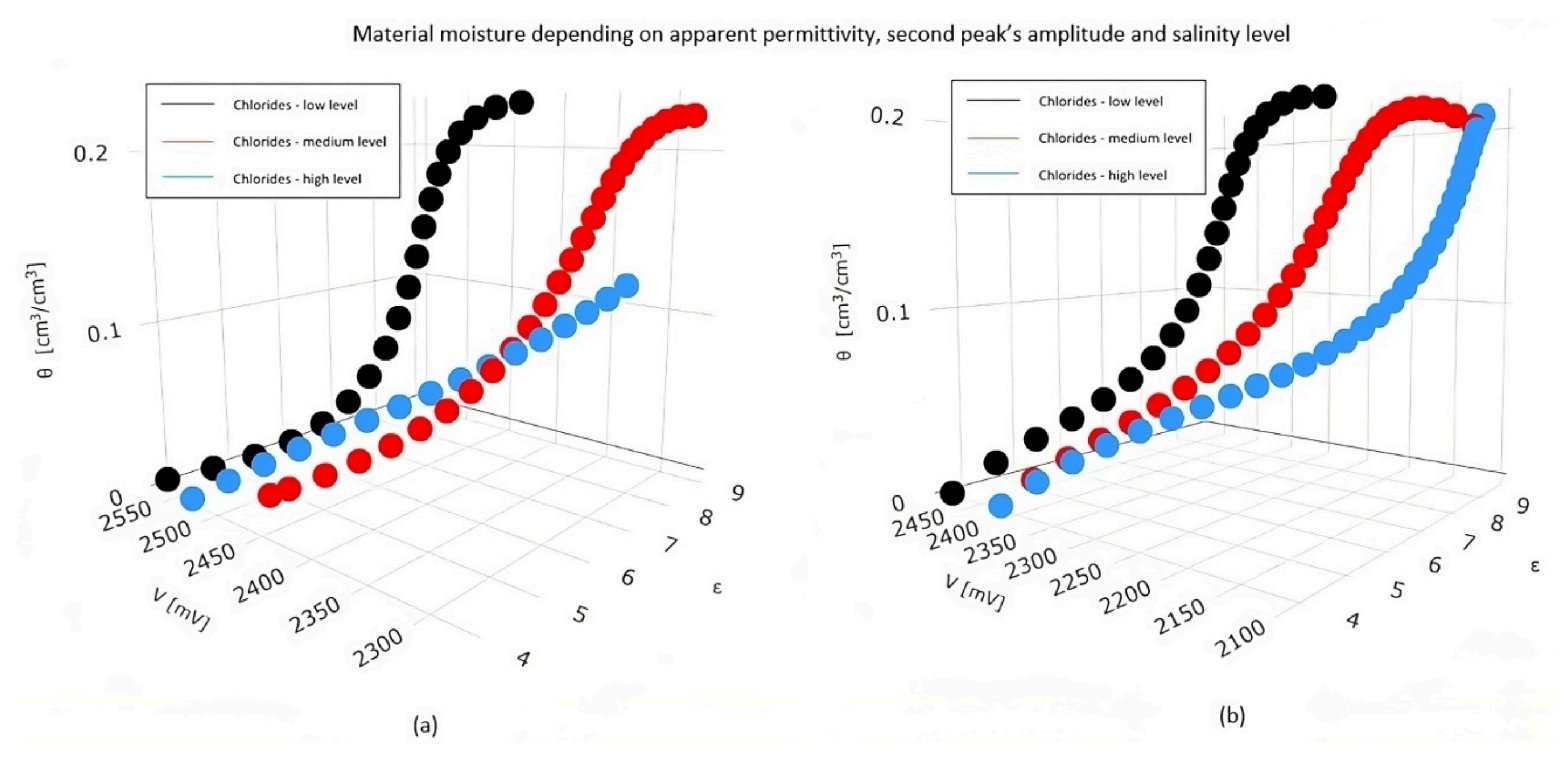
Moisture content (θ) related to permittivity (ε) and voltage (V) in red ceramic samples: (**a**) Wide Sensor, (**b**) Narrow Sensor.

**Figure 13:**
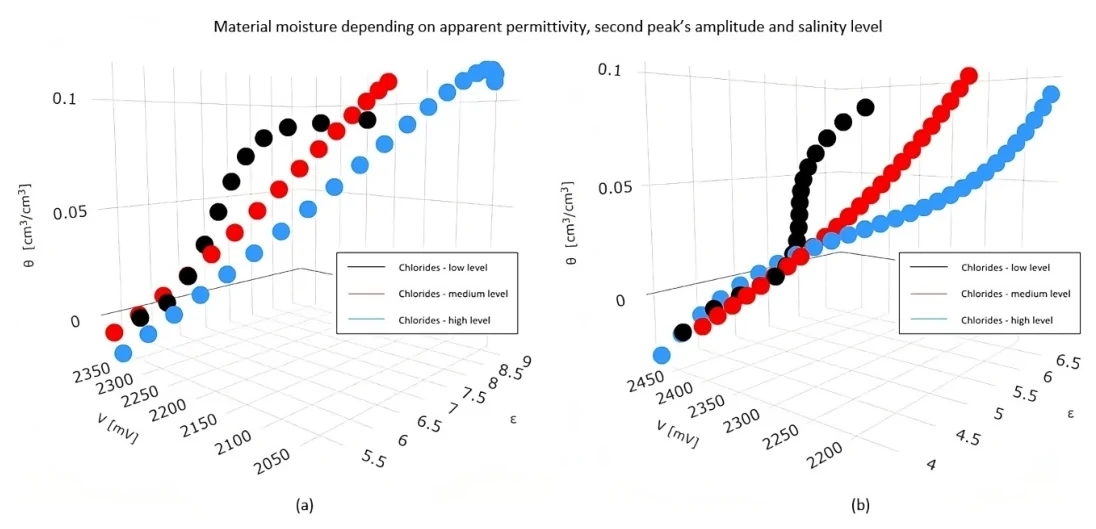
Moisture content (θ) related to permittivity (ε) and voltage (V) in clinker samples: (**a**) Wide Sensor, (**b**) Narrow Sensor.

**Figure 14:**
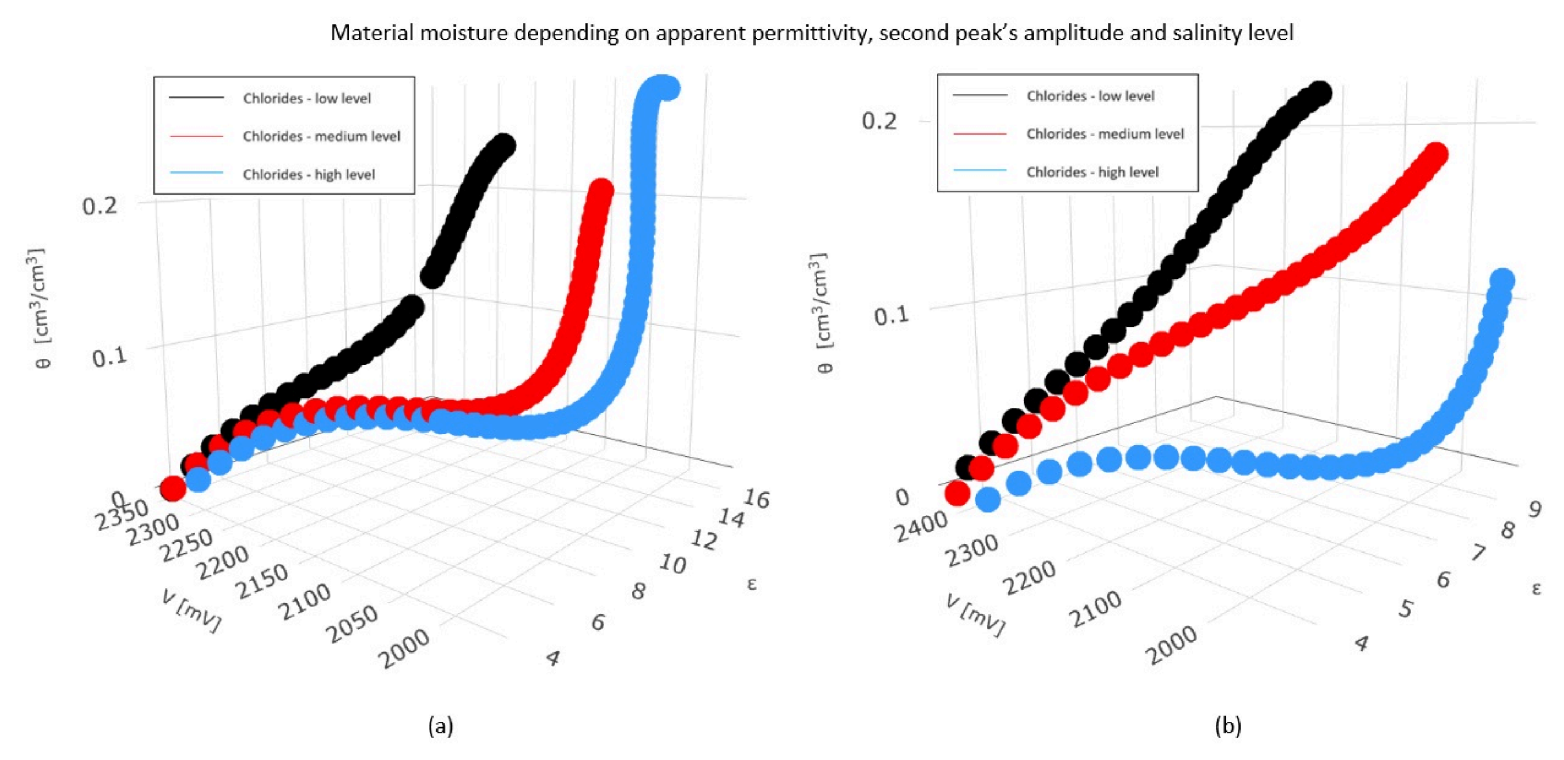
Moisture content (θ) related to permittivity (ε) and voltage (V) in silicate samples: (**a**) Wide Sensor, (**b**) Narrow Sensor.

The graphs in Figure 12, Figure 13 and Figure 14 present the dependence of volumetric water content on permittivity and second peak voltage values for all levels of chlorides (black—low level, red—medium level, blue—high level) for particular types of bricks. These plots were created using previously derived regression models (Table 7) based on data, the maximum ranges of which are included in Table 7.

More precisely, the diagrams in Figure 12, Figure 13 and Figure 14 represent the dependence between apparent permittivity and volumetric water content in the ε-θ plane, voltage and volumetric water content in the V-θ plane and finally the dependence between apparent permittivity and voltage for all levels of chlorides in the ε-V plane. In all diagrams, it can be seen that with moisture increase, apparent permittivity increases whereas the second peak’s voltage values decrease.

### 4.5. Comparison of Regression Models of One and Two Variables

To compare the quality of all approaches to modeling volumetric water content using the TDR signal, the following parameters were discussed: RMSE, RSE, R^2^/R^2^_adj_. For single-variable polynomial models θ(ε)_gen_ and θ(ε)_ind_, the averaged values of the fitting parameters of three levels of salinity were estimated and compared with multivariable polynomial models θ(ε,V). All of them are presented in Table 8.

The statistical significance of differences among the three regression approaches was additionally assessed using the non-parametric Friedman test. Statistically significant differences were found for R^2^ (*p* = 0.011), RMSE (*p* = 0.009), and RSE (*p* = 0.032). These results confirm that the choice of regression approach significantly affects the quality of model fitting and prediction accuracy. In general, the individual calibration models θ(ε)_ind_ and the multiple regression models θ(ε,V) provided better fitting performance than the general calibration model θ(ε)_gen_, as indicated by higher R^2^ values and lower RMSE and RSE values. Thus, the observed differences in model performance are supported by statistical evidence and cannot be attributed solely to random variation.

On the basis of R^2^/R^2^_adj_, the quality of multiple regression models θ(ε,V) is better than that of both types of regression models of one variable for the ceramic brick. For the silicate brick, the case is analogous, but for the clinker brick, the R^2^ of multiple regression models is worse compared to that of the individual θ(ε)_ind_ but better than that of the general θ(ε)_gen_ models. The average values of all coefficients of determination vary insignificantly (by 0.006) between the θ(ε)_ind_ and θ(ε,V) models and are significantly better than the θ(ε)_gen_ model. According to the RMSE values, the multiple regression models are better than the single individual regression models θ(ε)_ind_ for ceramic bricks (both types of sensors) and are slightly worse in other cases, but still significantly lower than for the general θ(ε)_gen_ model. The average RMSE values vary slightly (by 0.002 cm^3^/cm^3^) between the θ(ε)_ind_ and θ(ε)_ind_ models and are lower compared to the θ(ε)_gen_ general model. Taking into account the RSE values, the multiple regression models are also better than the single-variable regression models for the ceramic bricks in the case of both types of sensors. For clinker brick (Narrow Sensor) and silicate brick (Wide Sensor), the RSE values are exactly the same, and in other cases, they are slightly worse. The average RSE values differ very insignificantly (by 0.001 cm^3^/cm^3^) and are lower (better) than the RSE values achieved for general θ(ε)_gen_ models.

**Figure 15:**
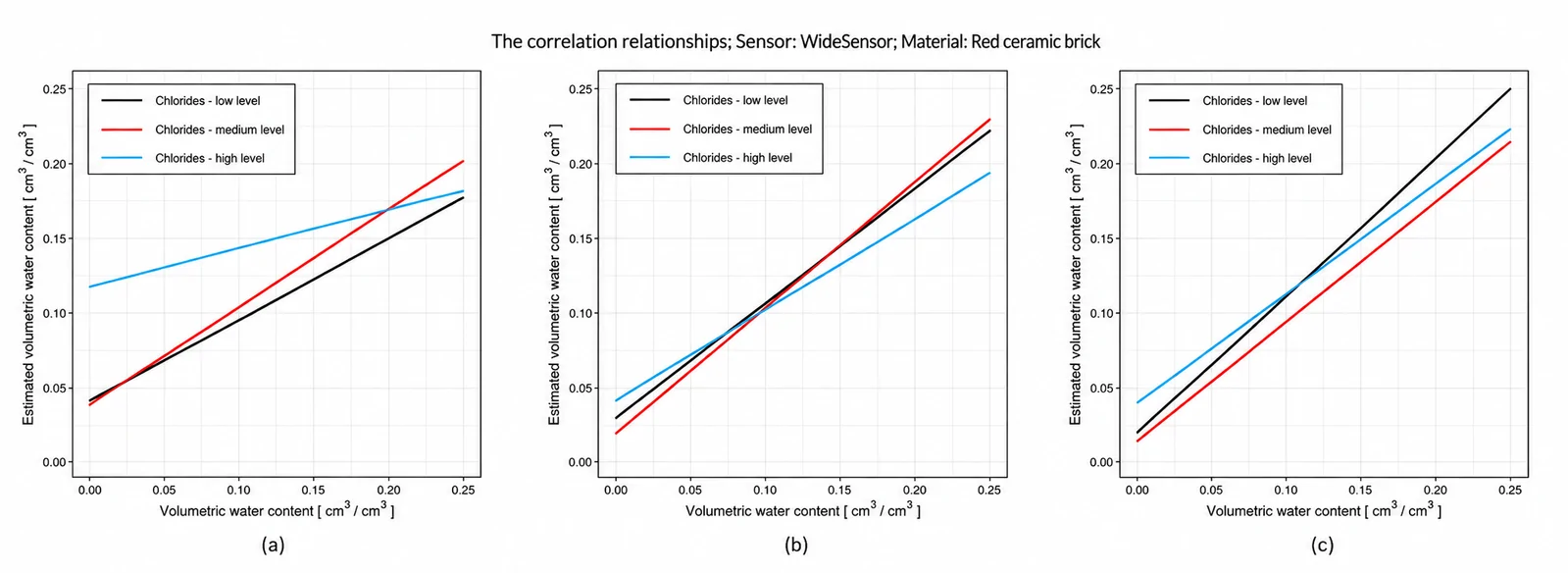
Correlation between the measured and estimated volumetric water content in red ceramic samples using the Wide Sensor: (**a**) θ and θ(ε)_gen_, (**b**) θ and θ(ε)_ind_, (**c**) θ and θ(ε,V).

**Figure 16:**
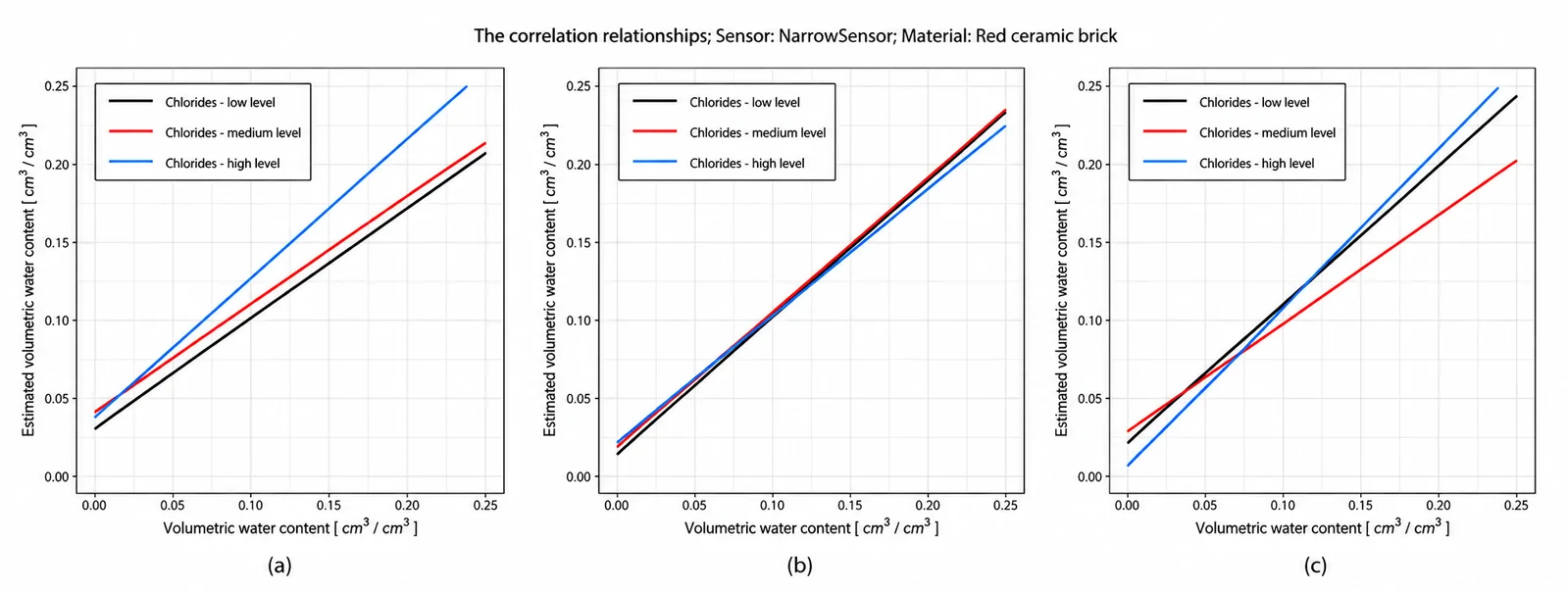
Correlation between the measured and estimated volumetric water content in red ceramic samples using the Narrow Sensor: (**a**) θ and θ(ε)_gen_, (**b**) θ and θ(ε)_ind_, (**c**) θ and θ(ε,V).

For comparative purposes, the correlations between the estimated values of volumetric water content achieved with the general θ(ε)_gen_ model, individual θ(ε)_ind_ models, the multivariable θ(ε,V) model and the volumetric water content values θ from the laboratory measurements were analyzed. These dependences are presented in the following order in the figures below. Firstly, the dependences for red ceramic brick and the wide TDR sensor are presented (Figure 15). The left diagram presents the correlation between θ(ε)_gen_ and θ values. The middle diagram shows the dependence between θ(ε)_ind_ and θ, and finally, the right one shows the dependence between θ(ε,V) and θ achieved in the laboratory. Figure 16 shows the same relationships for red brick and the Narrow Sensor.

It can be noticed from the diagrams that, for the general dependence θ(ε)_gen_, utilizing only the time approach for all levels of salinity, the lines are located far from the best-fit line, especially in the case of high chloride levels. On the other hand, individual θ(ε)_ind_ models show the best fit between laboratory and estimated moisture values. In the case of the θ(ε,V) models, the lines are more dispersed, but are much closer to the line of the greatest fit compared to the general models, with the only exception being for medium levels of chloride concentration, where this trend is not fulfilled. Table 9 presents linear regression estimators that are used for further comparison of the three presented methods, and averaged values of slope coefficients and y-intercept estimators are presented in Table 10.

In the case of the red ceramic brick in the multiple regression models θ(ε,V), the average values of the slope coefficients are closer to 1 than in the single regression θ(ε)_gen_ and in the individual θ(ε)_ind_ models, cf. Table 10. Moreover, the y-intercepts in the multiple regression models are closer to 0 than in the single regression models of both types. This confirms that the multiple regression models for ceramic brick for both types of sensors are better fitted compared to the general model that utilizes only time in apparent permittivity evaluation.

**Figure 17:**
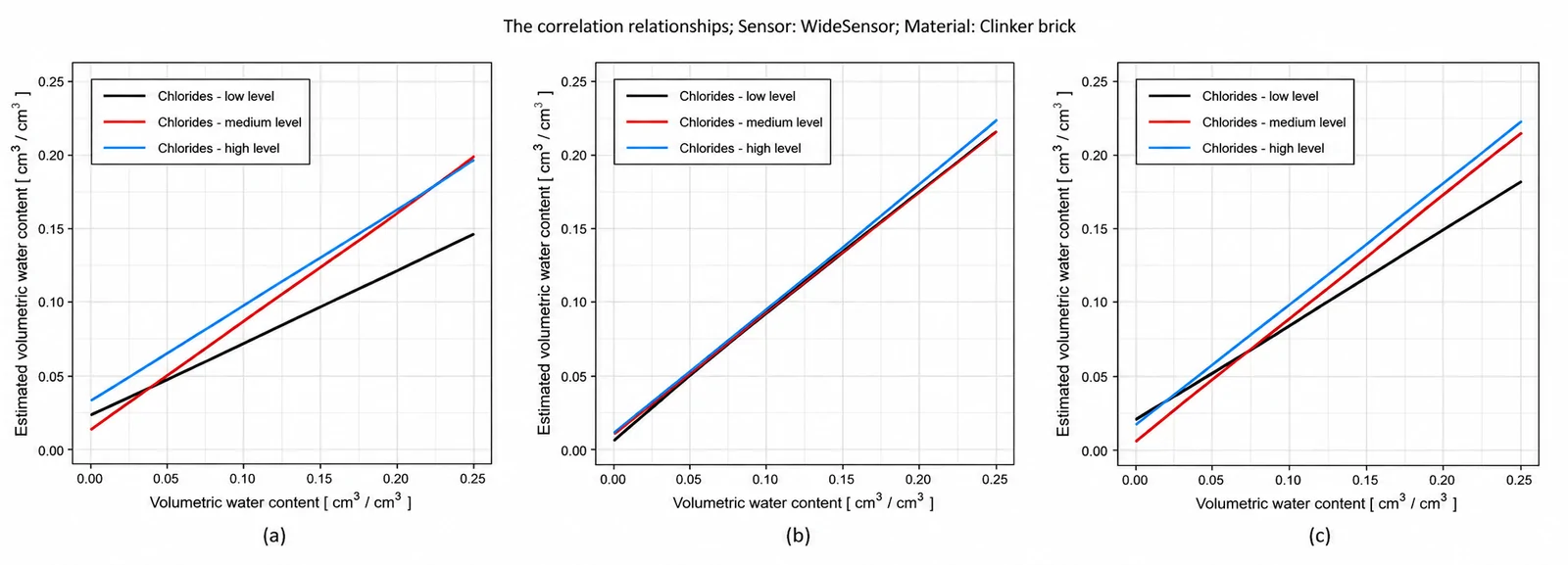
Correlation between the measured and estimated volumetric water content in clinker samples using the Wide Sensor: (**a**) θ and θ(ε)_gen_, (**b**) θ and θ(ε)_ind_, (**c**) θ and θ(ε,V).

**Figure 18:**
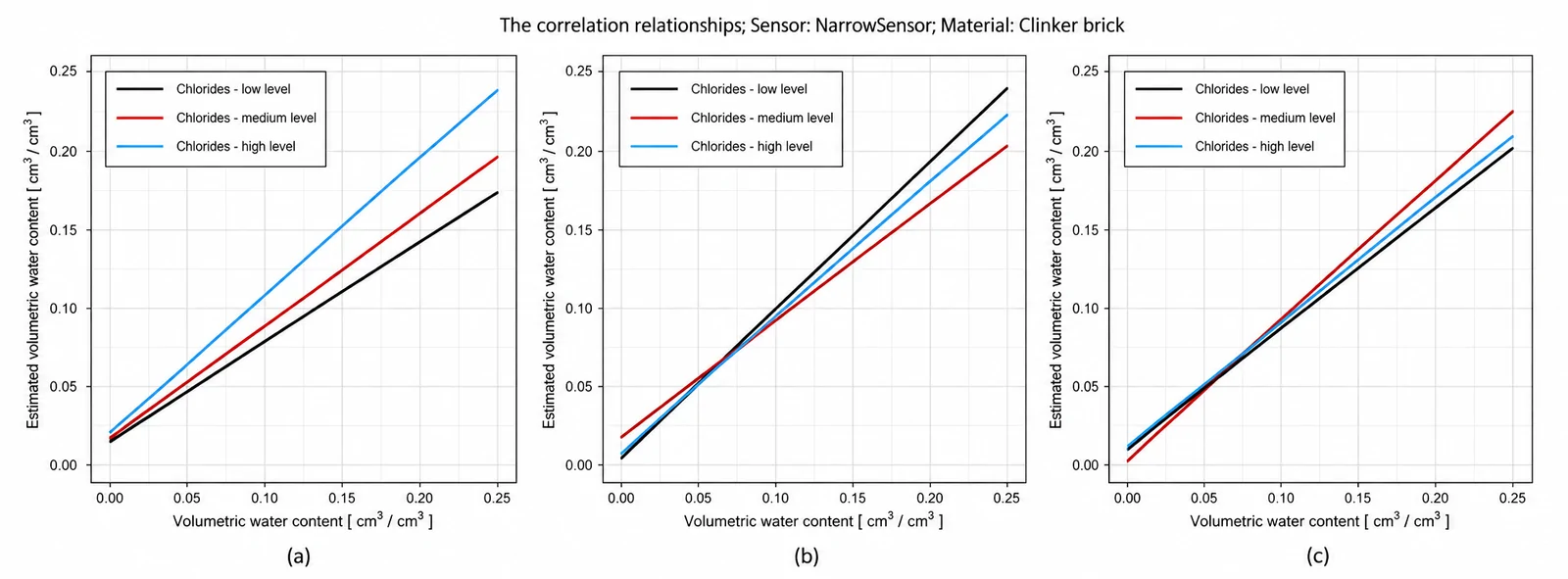
Correlation between the measured and estimated volumetric water content in clinker samples using the Narrow Sensor: (**a**) θ and θ(ε)_gen_, (**b**) θ and θ(ε)_ind_, (**c**) θ and θ(ε,V).

Similar analyses were conducted for both types of formulas for clinker brick—Figure 17 for the Wide Sensor and Figure 18 for the Narrow Sensor.

Table 11 presents correlation equations for clinker brick corresponding to the data gathered in Table 2 and Table 3, with low, medium, and high salinity levels, respectively.

**Table 11:** The equations of correlation for clinker brick related to Table 2 and Table 3 (L—low, M—medium, H—high).

Type of Sensor	Salinity Level	The Correlation Equations for θ(ε)gen. and θ	The Correlation Equations for θ(ε) and θ	The Correlation Equations for θ(ε,V) and θ
Wide Sensor	L	θ(ε) = 0.6958·θ + 0.0079	θ(ε) = 0.9337·θ + 0.0018	θ(ε,V) = 0.7665·θ + 0.0111
	M	θ(ε) = 0.8155·θ + 0.0085	θ(ε) = 0.9176·θ + 0.0031	θ(ε,V) = 0.8333·θ + 0.0072
	H	θ(ε) = 0.9635·θ + 0.0085	θ(ε) = 0.9431·θ + 0.0035	θ(ε,V) = 1.0416·θ − 0.0005
Narrow Sensor	L	θ(ε) = 0.7290·θ + 0.0075	θ(ε) = 0.9445·θ + 0.0043	θ(ε,V) = 0.8921·θ − 0.0006
	M	θ(ε) = 0.8600·θ + 0.0046	θ(ε) = 0.8864·θ + 0.0057	θ(ε,V) = 0.9431·θ − 0.0021
	H	θ(ε) = 0.9316·θ + 0.0162	θ(ε) = 0.9286·θ + 0.0024	θ(ε,V) = 0.9104·θ + 0.0008

**Figure 19:**
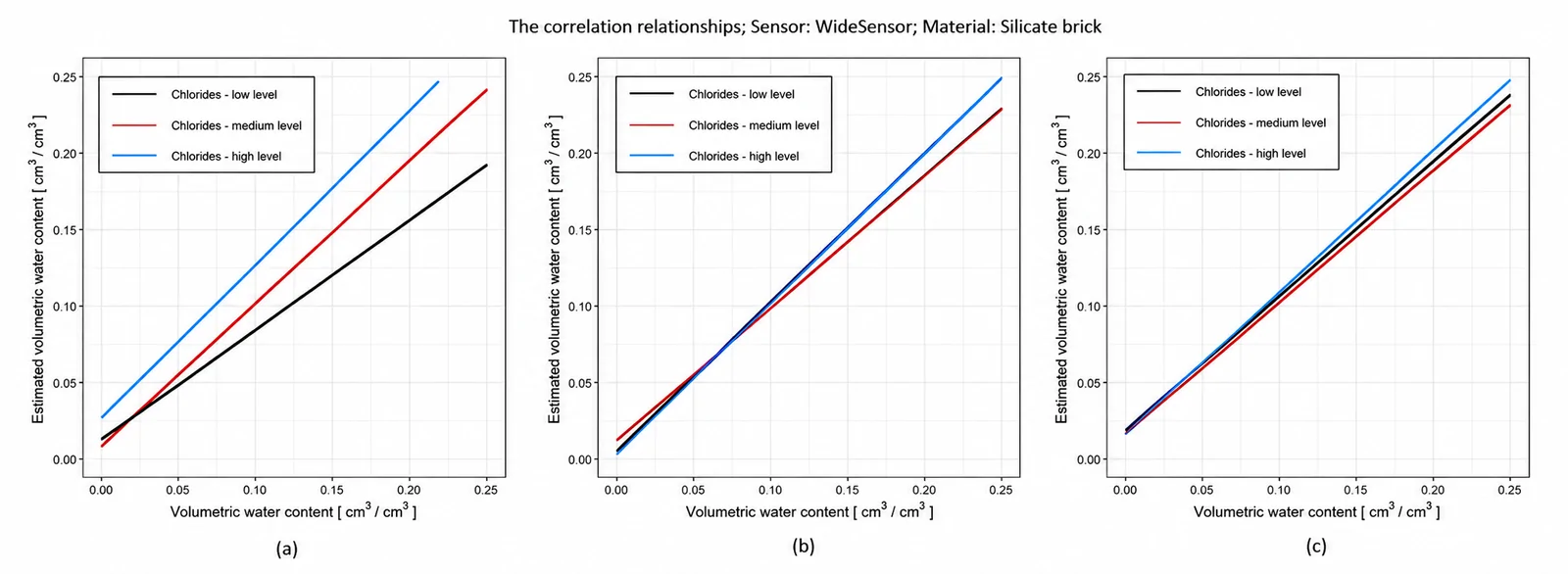
Correlation between the measured and estimated volumetric water content in silicate samples using the Wide Sensor: (**a**) θ and θ(ε)_gen_, (**b**) θ and θ(ε)_ind_, (**c**) θ and θ(ε,V).

**Figure 20:**
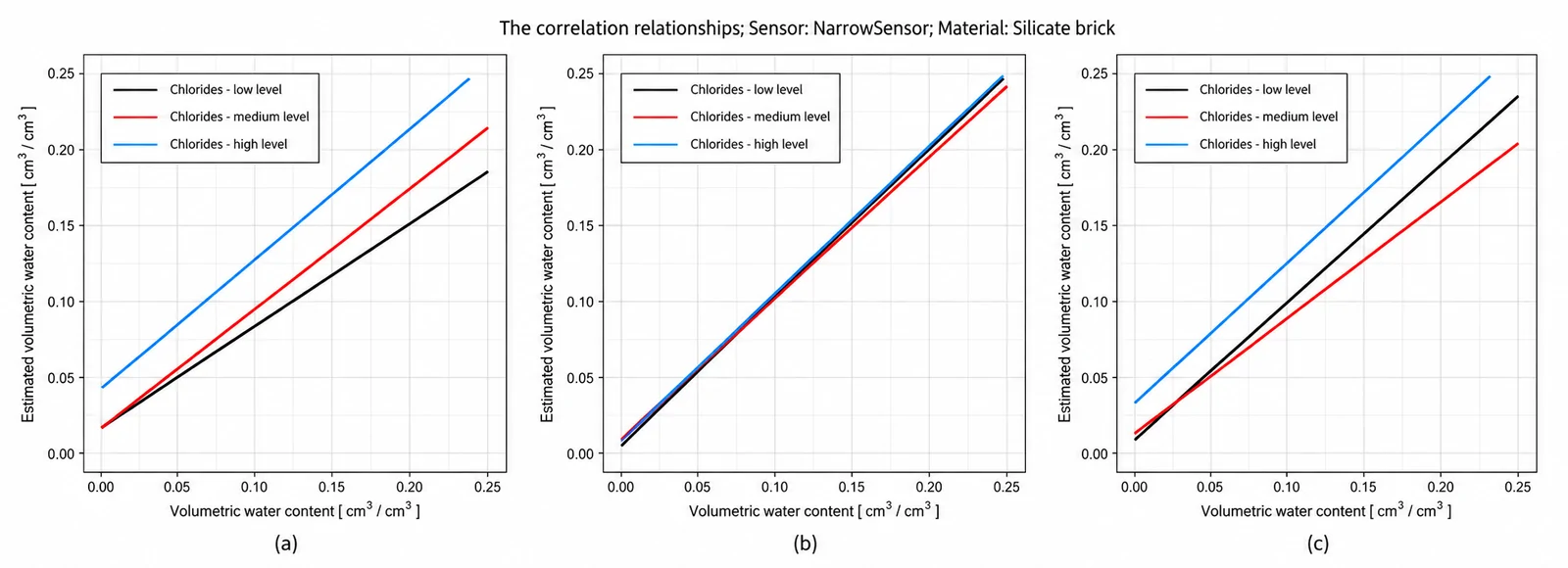
Correlation between the measured and estimated volumetric water content in silicate samples using the Narrow Sensor: (**a**) θ and θ(ε)_gen_, (**b**) θ and θ(ε)_ind_, (**c**) θ and θ(ε,V).

Finally, a similar correlation analysis was conducted for the silicate brick formulas to reveal the dependences between θ(ε)_gen_ and θ (left), θ(ε)_ind_ and θ (middle), and θ(ε,V) and θ (right). Figure 19 shows the correlation dependence for the Wide Sensor, and Figure 20 shows the result for the Narrow Sensor.

In the case of the clinker brick and the multivariable θ(ε,V) regression models, the average values of the slope coefficients are closer to 1 compared to the values achieved in general models θ(ε)_gen_. They are a little bit worse when compared to the slopes for individual models θ(ε)_ind_, but the difference is insignificant, cf. Table 12. Y-intercepts in all dependences are not large, but again, in the multiple regression models, similarly to the individual θ(ε)_ind_ models, they are closer compared to the traditionally achieved general models θ(ε)_gen_.

**Table 12:** The average values of correlation coefficients for clinker brick related to Table 11.

Type of Sensor	The Correlation Equations for					
	θ(ε)_gen_ and θ		θ(ε)_ind_ and θ		θ(ε,V) and θ	
	The Slope Coefficient	Y- Intercept	The Slope Coefficient	Y- Intercept	The Slope Coefficient	Y- Intercept
Wide Sensor	0.825	0.008	0.931	0.003	0.880	0.006
Narrow Sensor	0.840	0.009	0.920	0.004	0.915	−0.001

Table 13 presents correlation equations for silicate brick corresponding to the data presented in Table 2 and Table 3, with low, medium, and high salinity levels, respectively.

**Table 13:** The equations of correlation for silicate brick related to Table 2 and Table 3 (L—low, M—medium, H—high).

Type of Sensor	Salinity Level	The Correlation Equations for θ(ε)gen and θ	The Correlation Equations for θ(ε)ind and θ	The Correlation Equations for θ(ε,V) and θ
Wide Sensor	L	θ(ε) = 0.7168·θ + 0.0135	θ(ε) = 0.9950·θ + 0.0024	θ(ε,V) = 0.9448·θ + 0.0075
	M	θ(ε) = 0.9327·θ + 0.0089	θ(ε) = 0.8669·θ + 0.0126	θ(ε,V) = 0.9267·θ + 0.0055
	H	θ(ε) = 1.0076·θ + 0.0268	θ(ε) = 0.9967·θ + 0.0015	θ(ε,V) = 0.9893·θ + 0.0058
Narrow Sensor	L	θ(ε) = 0.6686·θ + 0.0188	θ(ε) = 1.0021·θ + 0.0013	θ(ε,V) = 0.9053·θ + 0.0087
	M	θ(ε) = 0.7866·θ + 0.0182	θ(ε) = 0.9314·θ + 0.0095	θ(ε,V) = 0.7629·θ + 0.0129
	H	θ(ε) = 0.8558·θ + 0.0444	θ(ε) = 1.0073·θ + 0.0025	θ(ε,V) = 1.0125·θ + 0.0184

In the case of the silicate brick and the Wide Sensor, the average values of the slope coefficients and the y-intercepts are very similar for the θ(ε)_ind_ individual models, cf. Table 14, while in the case of the general calibration model, these estimator values are worse.

**Table 14:** The average values of correlation coefficients for silicate brick related to Table 13.

Type of Sensor	The Correlation Equations for					
	θ(ε)_gen_ and θ		θ(ε)_ind_ and θ		θ(ε,V) and θ	
	The Slope Coefficient	Y- Intercept	The Slope Coefficient	Y- Intercept	The Slope Coefficient	Y- Intercept
Wide Sensor	0.886	0.016	0.953	0.006	0.954	0.006
Narrow Sensor	0.771	0.027	0.980	0.004	0.894	0.013

### 4.6. Discussion with the Literature

RMSE is an index providing information about the measuring quality of the method and was previously widely used by many scientists dealing with the TDR method; that is why it is discussed here in detail. In the case of moisture measurement of various materials, its value is difficult to discuss clearly because the sensors presented in many studies differ in construction; also, the tested materials have different water absorptivity and moisture ranges. Finally, some scientists present material moisture in different ways, not always in the form of volumetric water content; sometimes it is gravimetric or volumetric moisture, also expressed as a percentage. A comparison between RMSE values achieved in this research and found in the literature is presented in this paragraph. One of the most popular mixing formulas used for TDR calibration is the model proposed by Malicki et al. [56], where the RMSE for this formula is estimated to reach 0.03 cm^3^/cm^3^ and seems to be higher compared to the formulas developed within this research. Other recognized TDR scientists—Roth et al. [57]—give a wide range of RMSE values for the TDR method in their studies at the level of 0.008–0.037 cm^3^/cm^3^, depending on the type of tested material. In their research, Byun et al. [58] utilized a third-grade polynomial model to achieve RMSE values in the range between 0.04 and 0.05 cm^3^/cm^3^, depending on the material tested. Byun et al. [59] achieved another range of RMSE values for the TDR method, which was between 0.01 and 0.066 cm^3^/cm^3^. In their studies, Udawatta et al. [60] presented standard error values achieved for individual models to reach an RMSE between 0.008 and 0.034 cm^3^/cm^3^, and Domínguez-Niño et al. [61] have compiled RMSE values for different types of calibration models to reach lower values than achieved in the described research, ranging from 0.005 to 0.015 cm^3^/cm^3^. Ozgur [62] reported RMSE values for moisture evaluation in the range of 0.004–0.074, which were smaller than or similar to the values obtained here. Arsoy et al. [63] used Artificial Neural Networks to evaluate the soil water content to achieve values of 0.007–0.009 cm^3^/cm^3^, while standard calibration models were able to achieve RMSE values between 0.019 and 0.033 cm^3^/cm^3^ within the same research. Ankoye-Bempah et al. [64] used the TDR technique to evaluate the moisture of coffee beans and achieved an RMSE equal to 0.9% for a linear regression model between apparent permittivity and material moisture. Suchorab et al. [37] reported an RMSE range between 0.011 and 0.016 cm^3^/cm^3^ achieved under laboratory conditions for lightweight building materials.

The RMSE values described above concern invasive TDR probes, which are fully installed inside the tested material and are only dependent on its apparent permittivity. The sensors used in this research are noninvasive and are exposed to both the influence of the tested material and the ambient environment. This may lead to an increase in measurement errors. The co-authors of this article conducted many investigations using exactly these or similar sensors. In the previously mentioned article [37], Suchorab et al. achieved an RMSE value in the range between 0.024 and 0.032 cm^3^/cm^3^ depending on sensor construction. On the other hand, in article [26], similar sensors were applied, and the achieved RMSE value was equal to 0.013 cm^3^/cm^3^. In another article [36] dealing with noninvasive moisture detection using dielectric methods, RMSE values between 1.09 and 1.57% mass moisture were achieved, while in [18], they were estimated at 1.58–1.74% mass moisture for invasive probes and 1.5 and 2.7% for noninvasive probes, and the tested material was aerated concrete. Finally, noninvasive sensors were applied in the article by Mikusova et al. [16], where machine learning methods were used for moisture prediction and 2–2.4% of gravimetric moisture was achieved, in addition to values between 0.2 and 7.4% in the case of different machine learning methods.

When comparing the average values of R^2^/R^2^_adj_, RMSE, and RSE, as well as correlation formulas and their estimators, it could be stated that these indices are significantly better for both single individual θ(ε)_ind_ and multiple regression models θ(ε,V) than in the case of the typically applied general models θ(ε)_gen_. In many cases, it may be stated that the indices for θ(ε,V) are sometimes worse than for θ(ε)_ind_. On the other hand, it must be stated here that multiple regression models are both universal and provide measurement quality coefficients comparable to the individual calibration methods. With these models, the information about material salinity could be ignored, and the multiple regression model would take into account the presence of salt automatically. The presented single-variable regression equations θ(ε)_ind_ concerned materials with a known, laboratory-determined degree of salinity; therefore, they were precise. Multiple regression equations allow for the estimation of volumetric water content in saline media without prior knowledge of this parameter. This is a factor that influences the measurements and is difficult to estimate, requiring invasive tests. Multiple models are universal in nature, and yet in many cases they approach the parameters of detailed equations, sometimes exceeding them.

## 5. Conclusions

The research presented in this article concerns the possibility of improving the volumetric water content determination of building materials using the Time Domain Reflectometry method equipped with noninvasive sensors. The results showed that multiple regression, taking into account both the shift of measurement peaks in time and the attenuation of the signal amplitude in media with non-zero ionic conductivity, enables improvement of the measurement quality of the TDR method. The most important conclusion concerning the findings indicates that the proposed method of TDR sensor calibration provides satisfactory moisture evaluation in building partitions loaded with salt ions without the necessity to measure salinity levels. In the case of the red ceramic brick samples, the measurement quality expressed in RMSE and RSE was better compared to traditional methods, for which those values were lower, reaching 0.013 cm^3^/cm^3^ and 0.016 cm^3^/cm^3^, respectively. What is interesting is that even when compared to the individual formulas derived separately for different levels of salinity, the RMSE and RSE values were lower by 0.005 cm^3^/cm^3^ and 0.0045 cm^3^/cm^3^ in the case of the multiple regression models. For clinker and silicate brick samples, the RMSE and RSE values were comparable to individual calibration models, or sometimes slightly higher, but still significantly lower than the general formulas representing the traditional approach. Average RMSE values for both applied sensors and all tested materials were about 0.002 cm^3^/cm^3^ higher compared to the individual calibration models, but about 0.009 lower compared to the traditional method. Similarly, in terms of RSE, those differences were equal to 0.01 cm^3^/cm^3^ compared to the individual models, but 0.005 cm^3^/cm^3^ when taking into consideration the traditional approach. The general conclusion is that the multiple regression models proposed in the article estimate the moisture of materials similarly to the method that utilizes measurement of salinity levels, but significantly better compared to the traditional method. Finally, it should be noted that the RMSE values achieved using surface noninvasive sensors and derived multiple-regression calibration formulas are lower compared to many popular calibration formulas found in the current literature and utilized for standard, invasive probes.

The obtained results, which improve the quality of moisture estimation by considering the attenuation of the TDR signal caused by the presence of salt ions, are very promising. To achieve better prediction results and their validation, the following actions are planned: accounting for the presence of other salts in the tested materials and a more in-depth analysis of their other physical and chemical parameters; validation of the developed MLR models with the general Cole–Cole and Debye models, considering tests on materials from other production batches or manufacturers; as well as the development of universal models that would enable calibration of the device for most available materials. Additionally, we plan to implement regression machine learning models as an alternative method to improve the quality of estimation of building materials using the TDR method with varying salt ion contents.

## Figures and Tables

**Table 1:** Basic physical data of the used material samples.

Material	Average Density [kg/m^3^]	Average Mass of Dry Sample [g]	Average Mass of Saturated Sample [g]	Maximum Absorptivity [cm^3^/cm^3^]
Ceramic brick	1656	3100.2	3420.1	0.2254
Clinker brick	2320	4346.1	4456.74	0.1095
Silicate brick	1890	3457.1	3680.8	0.2353

**Table 2:** Regression models θ(ε)_gen_ established based on measurements of only the moisture level.

Material	Type of Sensor	Regression Model	R^2^	RSE [cm^3^/cm^3^]	RMSE [cm^3^/cm^3^]
Red ceramic brick	Narrow Sensor	θ(ε) = −0.006·ε^2^ + 0.1139·ε − 0.3345	0.802	0.027	0.031
	Wide Sensor	θ(ε) = −0.0075·ε^2^ + 0.1355·ε − 0.4222	0.704	0.031	0.038
Clinker brick	Narrow Sensor	θ(ε) = −0.005·ε^2^ + 0.0971·ε − 0.3201	0.835	0.015	0.012
	Wide Sensor	θ(ε) = −0.0103·ε^2^ + 0.1795·ε − 0.6772	0.835	0.015	0.013
Silicate brick	Narrow Sensor	θ(ε) = −0.0022·ε^2^ + 0.0582·ε − 0.1627	0.819	0.031	0.035
	Wide Sensor	θ(ε) = −0.0014·ε^2^ + 0.0465·ε − 0.1429	0.859	0.029	0.031

**Table 3:** Regression models θ(ε)_ind_ established based on measurements of moisture for different levels of salinity (L—low, M—medium, H—high).

Material	Type of Sensor	Salinity Level	Regression Model	R^2^	RSE [cm^3^/cm^3^]	RMSE [cm^3^/cm^3^]
Red ceramic brick	Narrow Sensor	L	θ(ε) = −0.005·ε^2^ + 0.116·ε − 0.3799	0.935	0.018	0.018
		M	θ(ε) = −0.0073·ε^2^ + 0.1434·ε − 0.4668	0.917	0.021	0.020
		H	θ(ε) = −0.0034·ε^2^ + 0.0728·ε − 0.2131	0.810	0.031	0.030
	Wide Sensor	L	θ(ε) = −0.0021·ε^2^ + 0.0903·ε − 0.3308	0.834	0.029	0.028
		M	θ(ε) = −0.008·ε^2^ + 0.1554·ε − 0.5249	0.895	0.023	0.023
		H	θ(ε) = −0.0046·ε^2^ + 0.0883·ε − 0.2602	0.627	0.043	0.043
Clinker brick	Narrow Sensor	L	θ(ε) = 0.0153·ε^2^ − 0.0963·ε + 0.1377	0.937	0.010	0.009
		M	θ(ε) = −0.0024·ε^2^ + 0.0712·ε − 0.2548	0.889	0.013	0.012
		H	θ(ε) = 0.0049·ε^2^ − 0.0139·ε − 0.0307	0.940	0.010	0.009
	Wide Sensor	L	θ(ε) = 0.0214·ε^2^ − 0.2014·ε + 0.460	0.941	0.010	0.009
		M	θ(ε) = −0.0046·ε^2^ + 0.1104·ε − 0.472	0.926	0.011	0.010
		H	θ(ε) = −0.0038·ε^2^ + 0.0809·ε − 0.3257	0.938	0.010	0.009
Silicate brick	Narrow Sensor	L	θ(ε) = −0.0027·ε^2^ + 0.0801·ε − 0.2512	0.993	0.007	0.007
		M	θ(ε) = −0.0014·ε^2^ + 0.0532·ε − 0.1595	0.920	0.024	0.023
		H	θ(ε) = −0.0007·ε^2^ + 0.0337·ε − 0.1114	0.977	0.013	0.013
	Wide Sensor	L	θ(ε) = −0.0023·ε^2^ + 0.0694·ε − 0.2296	0.982	0.012	0.011
		M	θ(ε) = −0.0013·ε^2^ + 0.0432·ε − 0.1285	0.892	0.028	0.027
		H	θ(ε) = −0.0007·ε^2^ + 0.0318·ε − 0.1088	0.986	0.010	0.010

**Table 4:** Equations estimating second peak voltage levels taking into account the level of salinity (L—low, M—medium, H—high).

Material	Type of Sensor	Salinity Level	Regression Model	R^2^
Ceramic brick	Wide Sensor	L	V = −17.364ε^3^ + 317.08ε^2^ − 1913.4ε + 6222.3	0.5618
		M	V = −4.5415ε^3^ + 100.11ε^2^ − 738.1ε + 4125	0.6931
		H	V = −1.7419ε^3^ + 47.11ε^2^ − 435.59ε + 3607.5	0.9026
	Narrow Sensor	L	V = −15.383ε^3^ + 283.7ε^2^ − 1734.2ε + 5781.1	0.7993
		M	V = −4.971ε^3^ + 106.56ε^2^ − 766.85ε + 4034.2	0.7994
		H	V = −2.0707ε^3^ + 57.183ε^2^ − 514.93ε + 3626	0.9464
Clinker brick	Wide Sensor	L	V = −79.542ε^3^ + 1516.6ε^2^ − 9658.1ε + 22,818	0.7269
		M	V = 2.5304ε^3^ − 25.591ε^2^ − 84.678ε + 3163.5	0.9617
		H	V = 5.4797ε^3^ − 92.258ε^2^ + 376ε + 2136.7	0.9898
	Narrow Sensor	L	V = −221.81ε^3^ + 3340.4ε^2^ − 16,665ε + 29,918	0.9426
		M	V = −4.0697ε^3^ + 74,949ε^2^ − 511.95ε + 3515.1	0.9532
		H	V = −4.2724ε^3^ + 102.27ε^2^ − 836.39ε + 4463.9	0.9924
Silicate brick	Wide Sensor	L	V = −1.8354ε^3^ + 48.536ε^2^ − 428.37ε + 3370.4	0.9779
		M	V = −1.2744ε^3^ + 40.354ε^2^ − 416.53ε + 3411.1	0.9867
		H	V = −0.5993ε^3^ + 23.26ε^2^ − 283.67ε + 3075.7	0.9616
	Narrow Sensor	L	V = −3.5586ε^3^ + 77.033ε^2^ − 569.31ε + 3638	0.9815
		M	V = −0.926ε^3^ + 30.08ε^2^ − 325.4ε + 3217.1	0.9599
		H	V = −1.3545ε^3^ + 44.203ε^2^ − 456.24ε + 3452.4	0.9868

**Table 5:** Range of variability of permittivity and voltage obtained from the readouts.

Material	Type of Sensor	ε_min_–ε_max_	*V_min_–V_max_*
Ceramic brick	Wide Sensor	3.820–11.706	2169–2555
	Narrow Sensor	3.614–13.150	2049–2462
Clinker brick	Wide Sensor	5.286–9.554	2070–2362
	Narrow Sensor	4.067–6.755	2162–2441
Silicate brick	Wide Sensor	3.651–19.477	1979–2349
	Narrow Sensor	3.483–15.697	2019–2439

**Table 6:** Regression models and fitting parameters for all tested materials and sensor types.

Material	Type of Sensor	Regression Model	R^2^_adj_	RSE [cm^3^/cm^3^]	RMSE [cm^3^/cm^3^]
Ceramic brick	Wide Sensor	θ(ε,*V*) = −7.531 + 0.1718ε − 0.008308ε^2^ + 0.00534*V* − 0.000001019*V*^2^	0.885	0.024	0.023
	Narrow Sensor	θ(ε,*V*) = −7.254 + 0.1387ε − 0.006449ε^2^ + 0.00563V − 0.00000116V^2^	0.904	0.022	0.021
Clinker brick	Wide Sensor	θ(ε,*V*) = 2.343 + 0.2854ε − 0.01647ε^2^ − 0.00333V + 0.0000007993*V*^2^	0.873	0.013	0.013
	Narrow Sensor	θ(ε,*V*) = −9.566 − 0.06293ε + 0.0117ε^2^ + 0.00813 − 0.000001717V^2^	0.908	0.011	0.012
Silicate brick	Wide Sensor	θ(ε,*V*) = −10.24 + 0.06571ε − 0.001925ε^2^ + 0.00898*V* − 0.000002008*V*^2^	0.955	0.017	0.017
	Narrow Sensor	θ(ε,*V*)= −5.838 + 0.08789ε − 0.003263ε^2^ + 0.004725*V* − 0.000001001V^2^	0.887	0.027	0.026

**Table 7:** Ranges of apparent permittivity and voltage taking into account the level of salinity (L—low, M—medium, H—high).

Material	Type of Sensor	Salinity Level	*ε_min_–ε_max_*	*V_min_–V_max_*
Ceramic brick	Wide Sensor	L	3.9–7.5	2382.113–2552.812
		M	4.2–8.9	2284.006–2454.450
		H	3.9–6.9	2272.606–2521.914
	Narrow Sensor	L	3.7–7.7	2225.486–2469.218
		M	3.9–9.3	2120.411–2369.388
		H	3.7–9.7	2105.903–2398.707
Clinker brick	Wide Sensor	L	5.5–7.5	2134.219–2341.800
		M	5.3–8.1	2143.341–2372.574
		H	5.3–9.1	2032.280–2353.774
	Narrow Sensor	L	4.1–5.8	2351.800–2456.300
		M	4.0–6.3	2246.900–2406.000
		H	4.0–6.7	2166.000–2481.200
Silicate brick	Wide Sensor	L	3.8–10.4	2100.430–2342.742
		M	3.8–11.6	2002.056–2341.107
		H	3.8–16.6	1965.568–2300.744
	Narrow Sensor	L	3.6–8.6	2175.826–2420.802
		M	3.4–9.6	2046.167–2422.069
		H	3.4–9.6	1936.130–2358.933

**Table 8:** Comparison of fitting parameters of all types of regression models. θ(ε)—single-variable polynomial models, θ(ε,V)—multivariable polynomial models, type of sensor W—wide, N—narrow.

Material	Type of Sensor	R^2^/R^2^ adj			RMSE [cm^3^/cm^3^]			RSE [cm^3^/cm^3^]		
		θ(ε) gen.	θ(ε) ind, avg	θ(ε,V)	θ(ε) gen.	θ(ε) ind, avg	θ(ε, V)	θ(ε) gen.	θ(ε) ind, avg	θ(ε,V)
Ceramic brick	W	0.704	0.785	0.885	0.038	0.031	0.023	0.031	0.032	0.024
	N	0.802	0.887	0.904	0.031	0.023	0.021	0.027	0.023	0.022
Clinker brick	W	0.835	0.935	0.873	0.015	0.009	0.013	0.014	0.010	0.013
	N	0.835	0.922	0.908	0.015	0.010	0.012	0.014	0.011	0.011
Silicate brick	W	0.859	0.953	0.955	0.031	0.016	0.017	0.029	0.017	0.017
	N	0.819	0.963	0.887	0.035	0.014	0.026	0.031	0.015	0.027
Average ceramic brick		0.753	0.836	0.895	0.035	0.027	0.022	0.029	0.028	0.023
Average clinker brick		0.835	0.929	0.891	0.015	0.0095	0.0125	0.014	0.011	0.012
Average silicate brick		0.839	0.958	0.921	0.033	0.015	0.0215	0.030	0.016	0.022
Average all		0.809	0.908	0.902	0.028	0.017	0.019	0.024	0.018	0.019

**Table 9:** The correlation equations for red ceramic brick related to Table 2 and Table 3 (L—low, M—medium, H—high).

Type of Sensor	Salinity Level	The Correlation Equations for θ(ε)_gen_ and θ	The Correlation Equations for θ(ε)_ind_ and θ	The Correlation Equations for θ(ε,V) and θ
Wide Sensor	L	θ(ε) = 0.6278·θ + 0.0286	θ(ε) = 0.8368·θ + 0.0192	θ(ε,V) = 0.9166·θ + 0.0192
	M	θ(ε) = 0.6614·θ + 0.0380	θ(ε) = 0.8893·θ + 0.0117	θ(ε,V) = 0.7974·θ + 0.0137
	H	θ(ε) = 0.7910·θ + 0.0311	θ(ε) = 0.6106·θ + 0.0411	θ(ε,V) = 0.9466·θ + 0.0037
Narrow Sensor	L	θ(ε) = 0.7091·θ + 0.0311	θ(ε) = 0.9267·θ + 0.0067	θ(ε,V) = 0.8913·θ + 0.0207
	M	θ(ε) = 0.6897·θ + 0.0414	θ(ε) = 0.9224·θ + 0.0097	θ(ε,V) = 0.7050·θ + 0.0267
	H	θ(ε) = 0.9684·θ + 0.0248	θ(ε) = 0.8133·θ + 0.0215	θ(ε,V) = 1.1148·θ − 0.0097

**Table 10:** The average values of correlation coefficients for red ceramic brick related to Table 9.

Type of Sensor	The Correlation Equations for					
	θ(ε)_gen_ and θ		θ(ε)_ind_ and θ		θ(ε,V) and θ	
	The Slope Coefficient	Y- Intercept	The Slope Coefficient	Y- Intercept	The Slope Coefficient	Y- Intercept
Wide Sensor	0.693	0.033	0.779	0.024	0.887	0.012
Narrow Sensor	0.789	0.032	0.887	0.013	0.904	0.013

## Data Availability

The original contributions presented in this study are included in the article. Further inquiries can be directed to the corresponding author.
